# Human breath as a carbon dioxide source for organic synthesis

**DOI:** 10.1039/d6ra05851e

**Published:** 2026-09-18

**Authors:** Seiji Shirakawa

**Affiliations:** a Graduate School of Integrated Science and Technology, Nagasaki University 1-14 Bunkyo-machi Nagasaki 852-8521 Japan seijishirakawa@nagasaki-u.ac.jp

## Abstract

The capture and utilization of low-concentration CO_2_ as a C1 feedstock for organic synthesis have emerged as important research areas. Although atmospheric CO_2_ is an ideal carbon source for sustainable organic transformations, its extremely low concentration (0.04%) presents a significant challenge to the development of efficient CO_2_ conversion processes. In this context, we turned our attention to human breath as an alternative CO_2_ source, which contains approximately 4% CO_2_. The capture and utilization of CO_2_ from human breath is also expected to play an important role in sustaining life in space, where readily available carbon sources are scarce and exhaled CO_2_ represents a valuable renewable carbon resource for life-support systems. Herein, we demonstrate that human breath can serve as a practical and readily available CO_2_ source for the efficient synthesis of urea compounds. Furthermore, the prepared urea compounds proved to be effective co-catalysts for the CO_2_ fixation with epoxides under pure CO_2_.

## Abstract 2

Human breath can be utilized as a source of carbon dioxide for organic synthesis.

## Graphical abstract

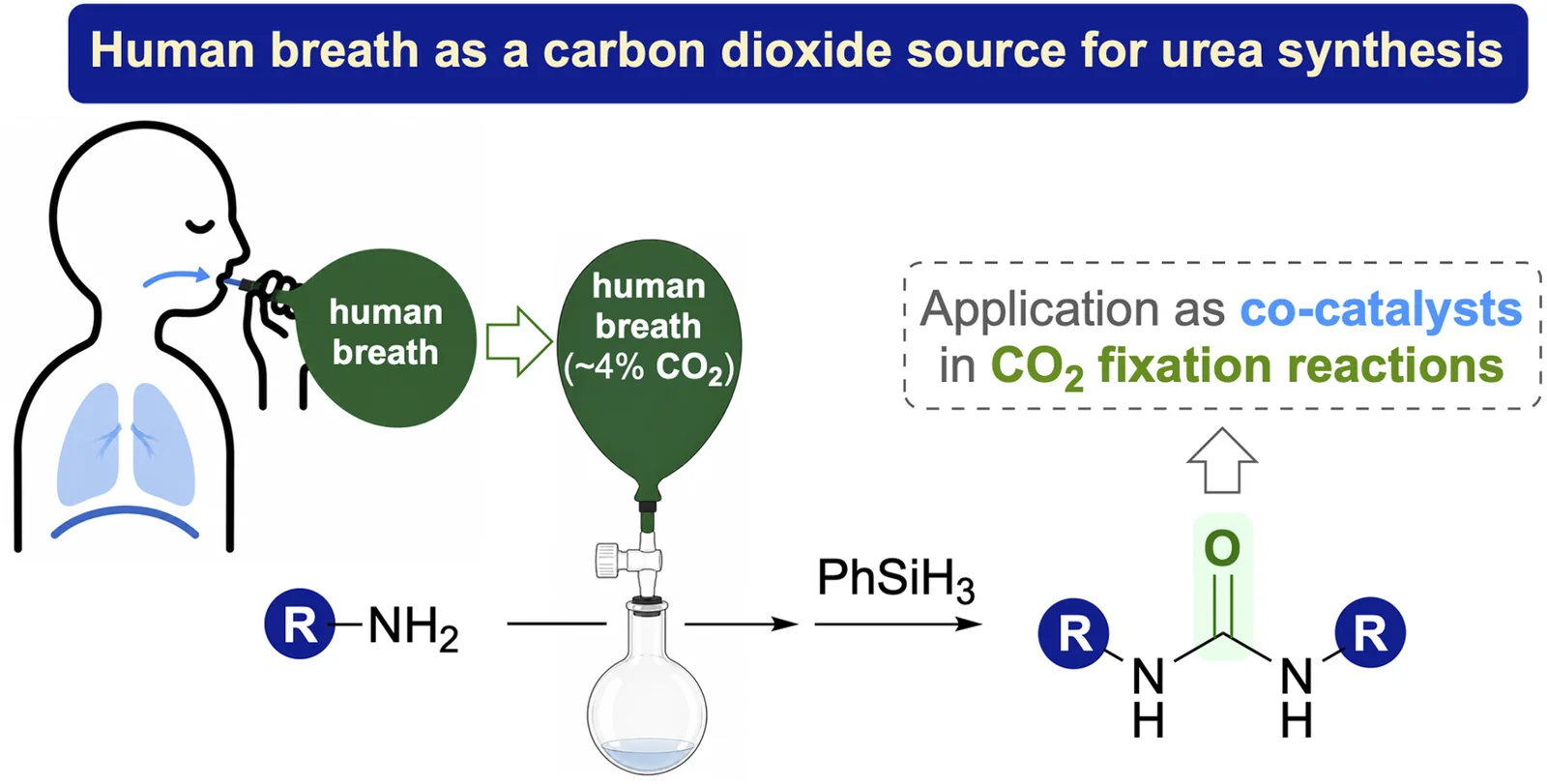

## Introduction

Transformation of carbon dioxide (CO_2_) into useful organic molecules has been among the most extensively studied topics in the field of green and sustainable chemical synthesis, particularly over the past two decades.^1–5^ The motivation for developing CO_2_ transformation reactions stems from the need to reduce atmospheric CO_2_ levels.^6–8^ To date, a wide variety of transformation reactions utilizing pure or highly concentrated CO_2_ have been developed. More recently, the capture and utilization of low-concentration CO_2_ as a C1 source for organic synthesis have emerged as important research areas.^9–13^ Industrial waste gases and ambient air are representative sources of low-concentration CO_2_. Among them, air is an ideal CO_2_ source for organic transformations because of its abundance and universal availability. However, the concentration of CO_2_ in air is only approximately 0.04% (∼400 ppm), and the efficient capture of such low-concentration CO_2_ remains one of the key technological challenges for achieving a sustainable society.^14–17^ Although air is the most readily available CO_2_ source for organic synthesis, its extremely low CO_2_ concentration makes the development of efficient CO_2_ transformation processes particularly challenging.

In this context, we became interested in human breath as an alternative CO_2_ source for organic synthesis (Scheme 1). Human breath contains approximately 4% CO_2_, which is about 100 times higher than the concentration found in air, making it a potentially convenient and accessible CO_2_ source for organic transformations.^18–21^ Additionally, the capture and utilization of CO_2_ from human breath has emerged as an important technology for sustaining life in space. In the space environment, where readily available carbon sources are limited, CO_2_ exhaled by humans represents a valuable carbon resource for maintaining life-support systems.^22^ Surprisingly, however, the utilization of human breath as a CO_2_ source for chemical synthesis has remained largely unexplored. Herein, we demonstrate that human breath can serve as a practical and readily available CO_2_ source for organic synthesis through the efficient preparation of urea compounds.^23^

**Scheme 1:**
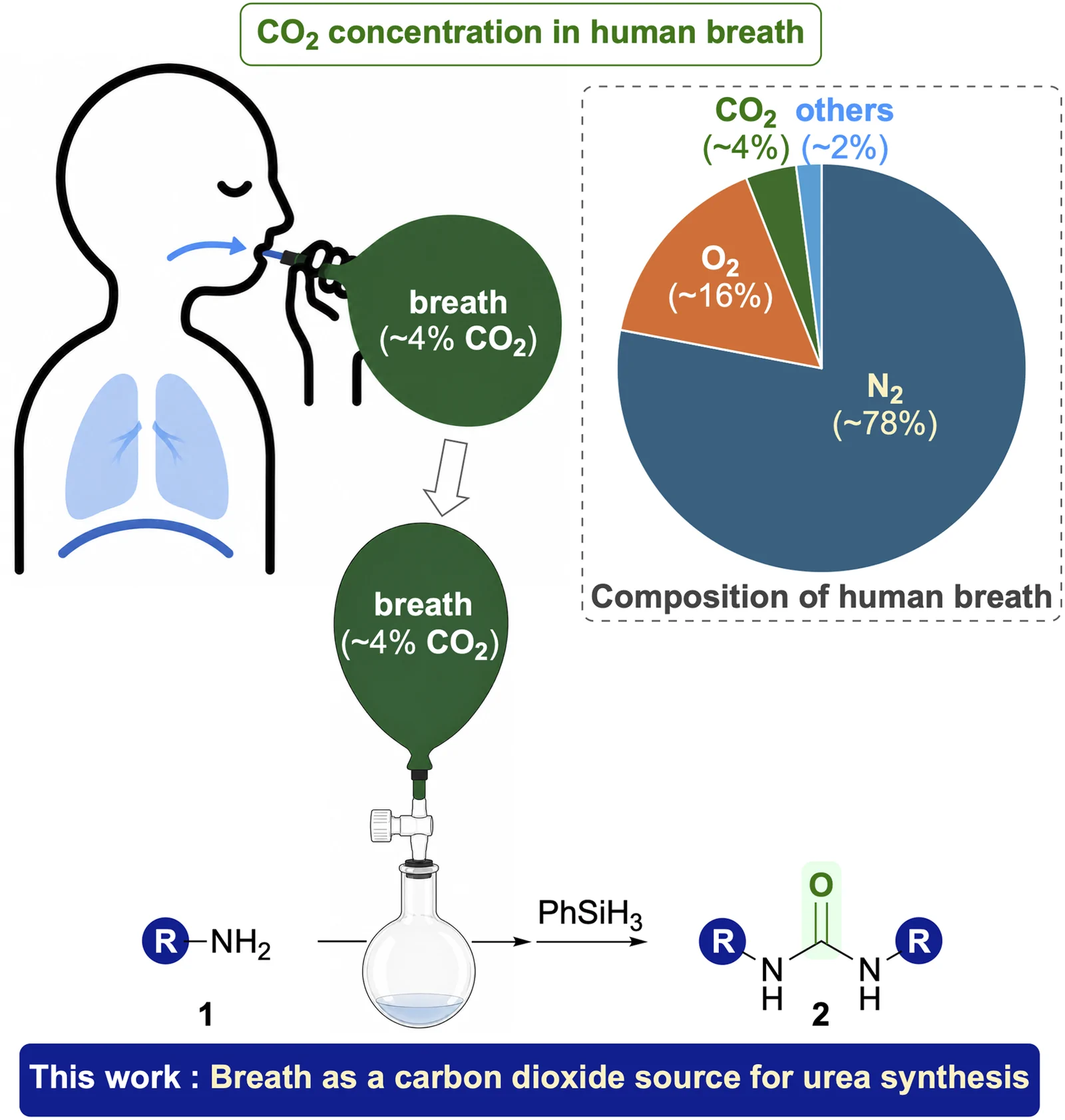
Human breath as a carbon dioxide source for organic synthesis.

## Results and discussion

### Human breath as a carbon dioxide source for urea synthesis

Our study began with the synthesis of dioctylurea 2a from octylamine 1a using human breath as a CO_2_ source (Scheme 2). Octylamine 1a was stirred at ambient temperature for 24 hours under a human breath atmosphere in a 2 L balloon. Phenylsilane was then added to the reaction flask.^24–26^ The resulting mixture was heated at 100 °C for 20 hours to afford the target dioctylurea 2a. To assess the reproducibility of the reaction, the reaction of octylamine 1a with human breath was performed three times. Dioctylurea 2a was obtained in consistently good yields (83–87%) in all three experiments under solvent-free conditions. Furthermore, even when the exposure time of octylamine 1a to human breath was reduced to 2 hours, dioctylurea 2a was still obtained in good yield (75%, Scheme S1, SI). The reaction using a commercially available gas mixture containing 10% CO_2_ in nitrogen afforded a comparable yield to that obtained under a human breath atmosphere (Scheme S2, SI). Although human breath generally contains a few percent of water vapor,^27–29^ the present synthetic method for urea compounds 2 is not significantly affected by the presence of water vapor under the reaction conditions. It is noteworthy that the reaction conducted in the absence of phenylsilane did not afford the corresponding urea product 2a (Scheme S3, SI). A plausible role of phenylsilane in the reaction of octylammonium octylcarbamate is proposed in Scheme S4 (SI).^26^

**Scheme 2:**
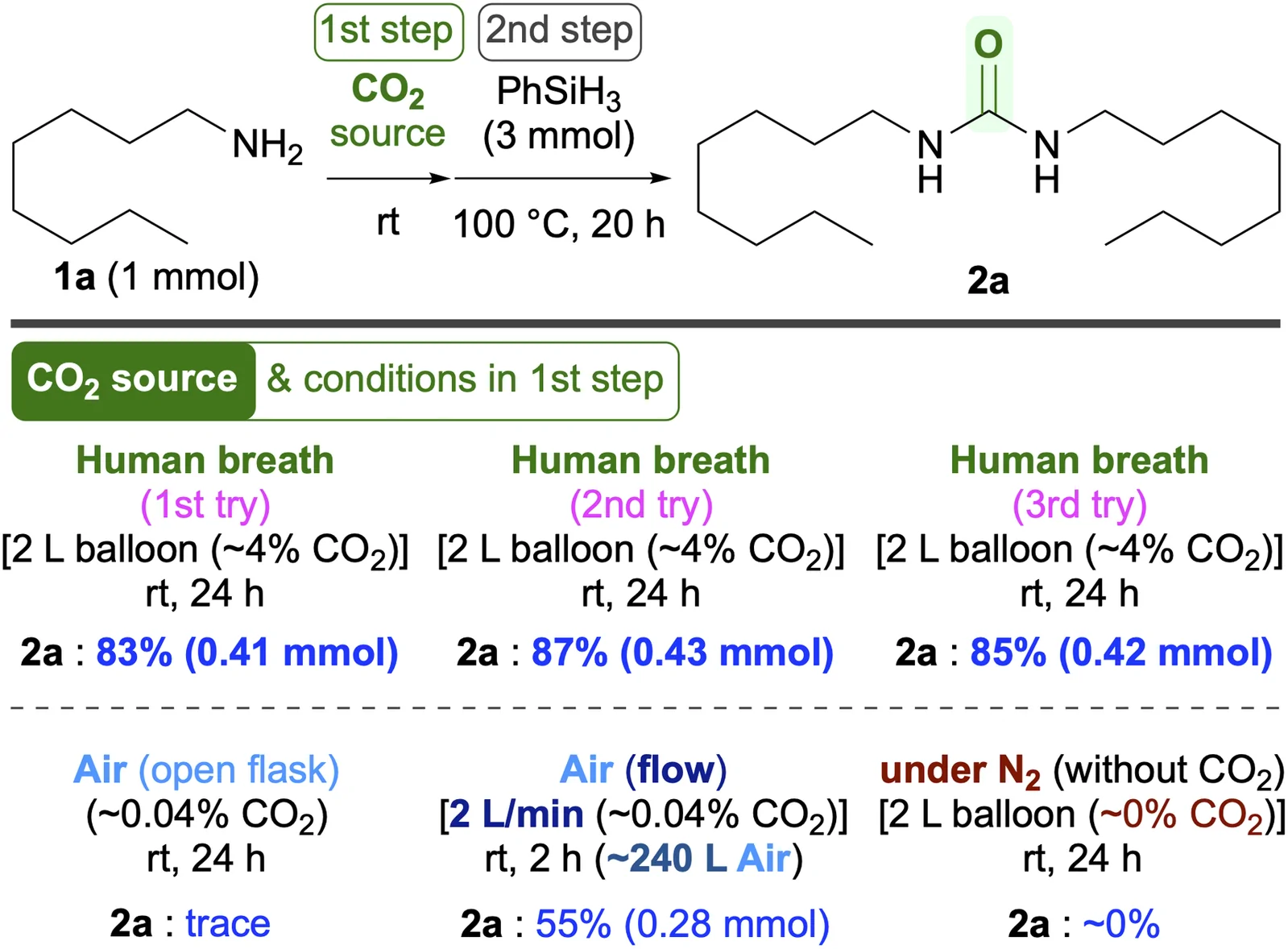
Urea synthesis from amine with low-concentration CO_2_.

The reaction of octylamine 1a was also examined under open-air conditions (Scheme 2). After stirring 1a in air for 24 hours, phenylsilane was added to the reaction mixture. Only trace amounts of dioctylurea 2a were detected in the crude reaction mixture by ^1^H NMR analysis. We also investigated the reaction under air-flow conditions. Approximately 240 L of air was passed through a flask containing octylamine 1a over 2 hours (2 L min^−1^). Subsequent treatment with phenylsilane afforded dioctylurea 2a in a moderate yield (55%). These results indicate that atmospheric CO_2_ can also serve directly as a carbon source in the present reaction system, although a large volume of air (240 L) was required to achieve a moderate yield. In contrast, no dioctylurea 2a was formed when the reaction was conducted under a nitrogen atmosphere. It should be noted that cyclic urea syntheses from diamines using a flow of 10–15% CO_2_ in nitrogen have previously been reported in metal-catalyzed systems employing sealed or autoclave reactors at relatively high reaction temperatures (140–170 °C) in polar solvents.^30–32^

To obtain direct evidence for the capture of CO_2_ from human breath by octylamine 1a, NMR experiments were conducted (Scheme 3). When octylamine 1a (a colorless liquid) was exposed to human breath under conditions essentially identical to those employed in the first step of the urea synthesis shown in Scheme 2 (room temperature, 24 hours), a white solid was formed, indicating the capture of CO_2_ from human breath by octylamine 1a (1a-CO_2_). The resulting solid was dissolved in CD_3_OD and analyzed by ^1^H and ^13^C NMR spectroscopy.

**Scheme 3:**
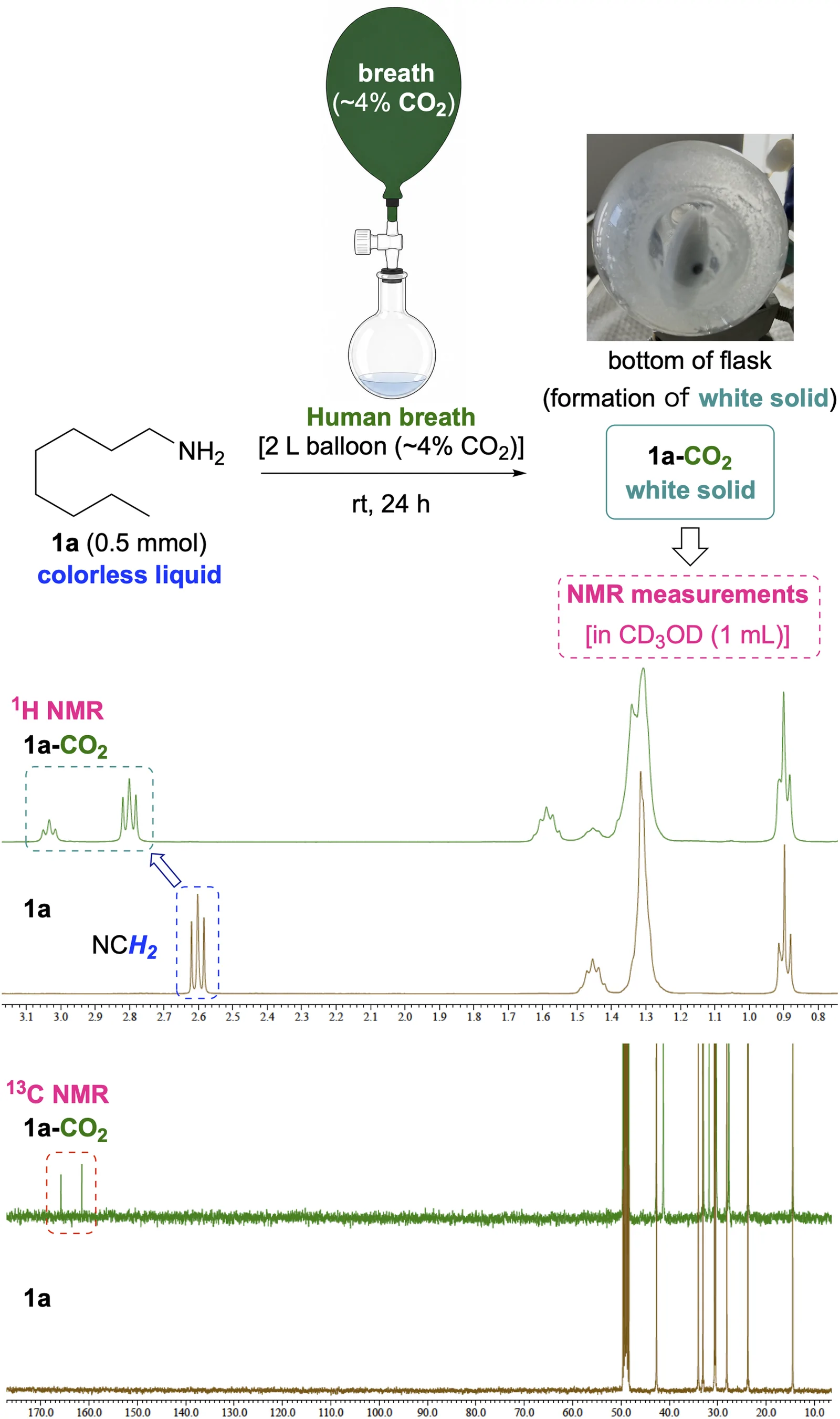
Absorption of CO_2_ in human breath by amine.

As shown in Scheme 3, the ^1^H NMR spectrum of 1a-CO_2_ exhibited clear downfield shifts of the alkyl proton signals compared with those of octylamine 1a. In particular, the methylene protons adjacent to the nitrogen atom (NCH_2_) showed a pronounced downfield shift. Notably, the observed ^1^H NMR spectrum of 1a-CO_2_ was very similar to that previously reported for the octylamine-CO_2_ adduct, which was identified as octylammonium octylcarbamate.^33^

Further evidence for the capture of CO_2_ from human breath by octylamine 1a was obtained from ^13^C NMR analysis shown in Scheme 3. The ^13^C NMR spectrum of 1a-CO_2_ in CD_3_OD exhibited distinct carbonyl carbon signals at 166 and 161 ppm, whereas no such signals were observed in the spectrum of octylamine 1a. These results strongly support the formation of a CO_2_-derived adduct upon exposure of octylamine 1a to human breath.^34–36^

The ^1^H and ^13^C NMR analyses shown in Scheme 3 provided direct evidence for the capture of CO_2_ from human breath by octylamine 1a (1a-CO_2_). Furthermore, two major species were detected in the NMR spectra of 1a-CO_2_ in CD_3_OD. Although the exact structures of the species observed for 1a-CO_2_ remain unclear at this stage, the two carbonyl signals observed in the ^13^C NMR spectrum are tentatively assigned to carbamic acid, carbamate, bicarbonate, and/or carbonate species based on previous reports,^34–36^ as shown in Scheme 4.

**Scheme 4:**
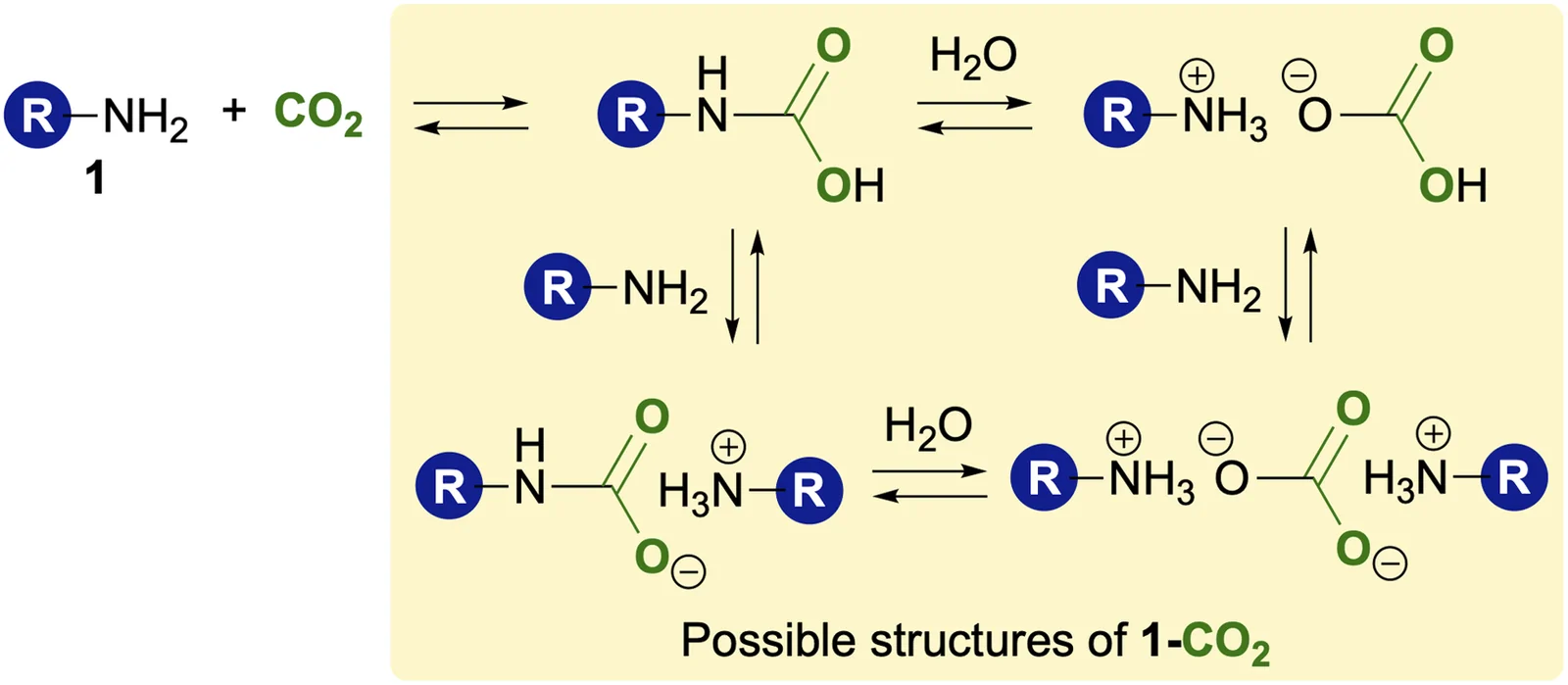
Structures of amine-CO_2_ adducts.

With direct evidence for the capture of CO_2_ from human breath by primary amines in hand, several important urea compounds were prepared using human breath as a CO_2_ source by the present method (Scheme 5). Normal and branched alkyl chain-substituted ureas 2a–c were obtained in good to excellent yields (76–97%) from the corresponding alkylamines 1a–c. When phenethylamine 1d was employed as the starting primary amine, the corresponding urea 2d, which is known as a metabolite of phenylalanine,^37,38^ was obtained in 80% yield. The related urea 2e was also obtained in high yield (92%) from phenylpropylamine 1e. Cyclooctylamine 1f was likewise compatible with the present urea synthesis, affording the corresponding product 2f in 56% yield. The steric hindrance around the nitrogen atom in 1f may account for the lower yield of 2f compared with those of the other urea products. The reaction of adamantanemethylamine 1g provided biologically active urea 2g in good yield (80%).^39,40^ Oxygen-containing urea compounds 2h and 2i were also prepared from the corresponding primary amines 1h and 1i in 69% and 98% yields, respectively. In addition, the present method was also applicable to the synthesis of cyclic ureas, affording *trans*-2j and *cis*-2j from *trans*-1j and *cis*-1j, respectively. On the other hand, the reaction of aniline did not afford the corresponding urea product under the present reaction conditions (Scheme S5, SI). Similar limitations in the scope of amine substrates have also been reported for the organocatalytic synthesis of ureas from primary amines under pure CO_2_ (100% CO_2_ gas).^26^

**Scheme 5:**
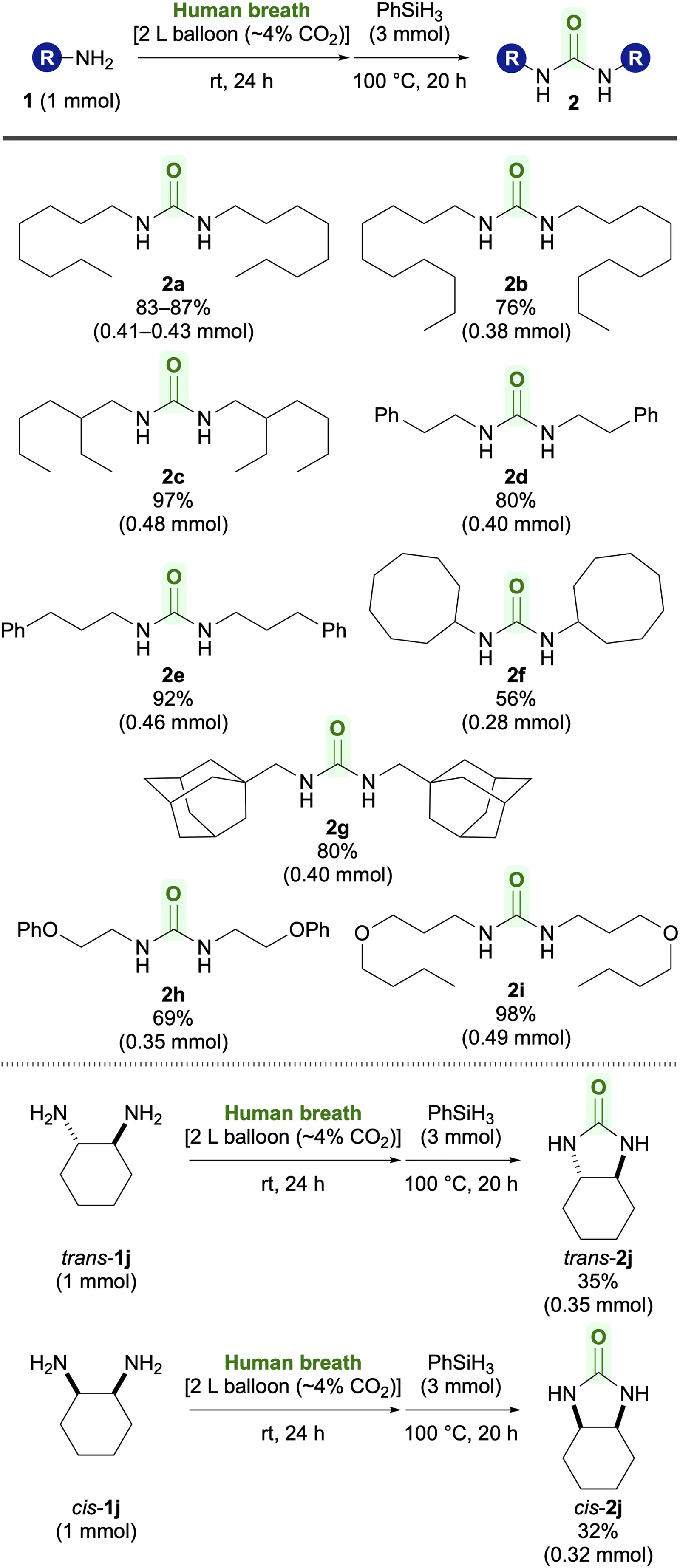
Urea synthesis from amines using human breath as a CO_2_ source.

### Utility of urea compounds as co-catalysts for the CO_2_ fixation reaction with epoxides under pure CO_2_

To demonstrate the utility of the prepared urea compounds 2, their ability to act as co-catalysts in the CO_2_ fixation reaction with epoxides 3, which is a representative rection for catalytic CO_2_ fixation,^41–46^ was investigated (Scheme 6). When styrene oxide 3a was treated with tetrabutylammonium iodide (5 mol%) under (pure) CO_2_ atmosphere at 40 °C for 24 hours, the CO_2_ fixation reaction proceeded only slowly, affording cyclic carbonate 4a in 19% yield. Urea compounds 2 (5 mol%) were then employed as co-catalysts to further promote the reaction. The addition of urea co-catalysts clearly accelerated the CO_2_ fixation with 3a, giving cyclic carbonate 4a in moderate yields (35–45%; see also the bottom of Scheme 6). Among the urea derivatives examined, urea 2i exhibited slightly higher catalytic activity than the other ureas, as shown in Scheme 6. Under optimized conditions employing tetrabutylammonium iodide and urea 2i (5 mol% each) at 60 °C for 24 hours under CO_2_ atmosphere, cyclic carbonate 4a was obtained in good yield (85%). It should be noted that the use of urea 2i alone, in the absence of tetrabutylammonium iodide, resulted in almost no promotion of the CO_2_ fixation reaction with styrene oxide 3a (bottom of Scheme 6).

**Scheme 6:**
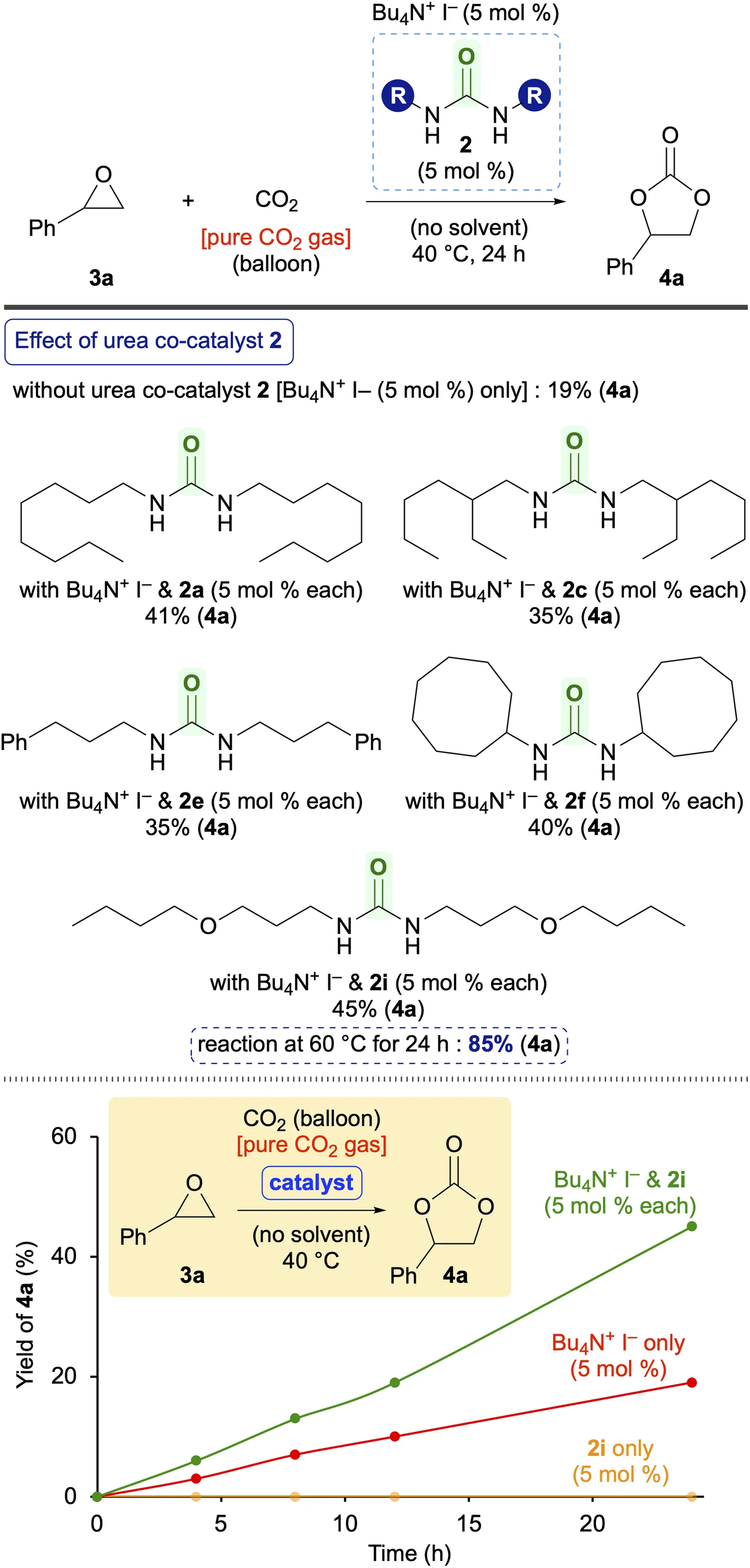
Utility of urea compounds as a co-catalyst in the CO_2_ fixation reaction.

The binary catalytic system consisting of tetrabutylammonium iodide and urea 2i as a co-catalyst (5 mol% each) efficiently promoted the CO_2_ fixation with epoxides 3 (Scheme 7).^47,48^ When the reactions were carried out at 60 °C for 24 hours under atmospheric CO_2_ pressure, the corresponding cyclic carbonates 4a–c were obtained in high yields (85–94%). The present binary catalytic system enabled solvent-free CO_2_ fixation under atmospheric pressure without the use of an autoclave and at a mild reaction temperature, thereby reducing energy consumption.

**Scheme 7:**
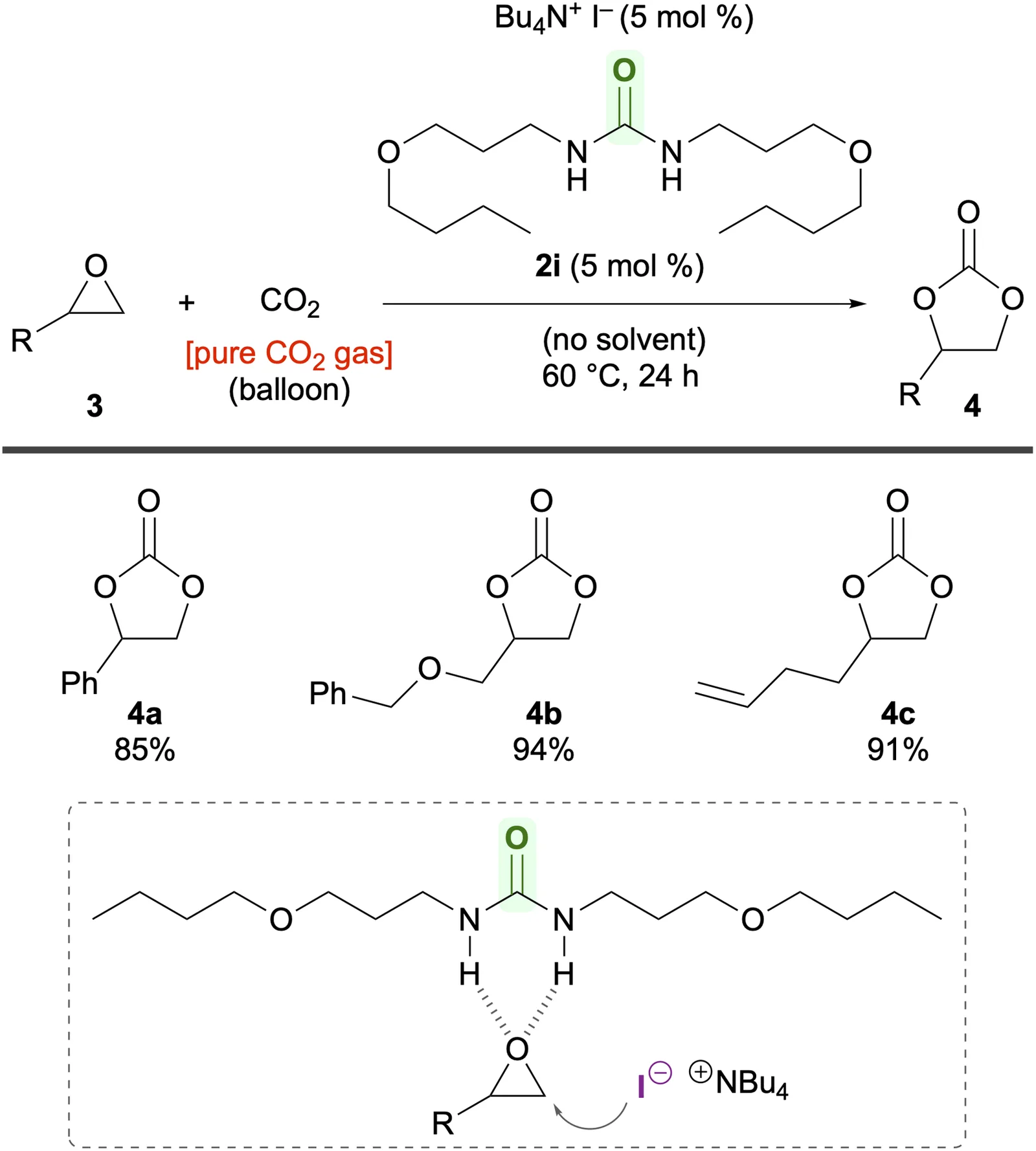
CO_2_ fixation reactions with a urea co-catalyst.

## Conclusions

We have successfully demonstrated that human breath can serve as a practical CO_2_ source for organic synthesis. Organo-urea compounds 2 were prepared from primary amines 1 under a human breath atmosphere and solvent-free conditions. When liquid primary amines 1 were exposed to human breath, white solids were formed as a result of CO_2_ capture by the amines, which was confirmed by NMR studies. Subsequent treatment of the resulting solid intermediates with phenylsilane afforded the desired organo-urea compounds 2. The present method provides a simple approach to the synthesis of organo-urea compounds from commercially available reagents using readily available human breath as a CO_2_ source under solvent-free conditions. The utility of the prepared urea compounds 2 as co-catalysts was demonstrated in the CO_2_ fixation reactions with epoxides 3 under pure CO_2_. In combination with tetrabutylammonium iodide, organo-urea compounds 2 acted as co-catalysts for the conversion of epoxides 3 into cyclic carbonates 4, providing the products in high yields. Further synthetic applications of human breath as a C1 source for the preparation of valuable organic compounds are currently under investigation in our research group.

## Ethics statement

All experiments involving human breath were performed in accordance with the Guidelines for Life Science and Medical Research Involving Human Subjects and were approved by the Ethics Committee at Graduate School of Integrated Science and Technology, Nagasaki University (Approval No. R080909IST-09). Informed consent was obtained from the participant who provided human breath for this study.

## Author contributions

S. S. conceived and supervised the project, performed the experiments, analyzed the data, and wrote the manuscript.

## Conflicts of interest

There are no conflicts to declare.

## Supplementary Material

RA-016-D6RA05851E-s001

## Data Availability

The data supporting this article have been included as part of the supplementary information (SI). Supplementary information is available. See DOI: https://doi.org/10.1039/d6ra05851e.
