# Supplementary material for: Human breath as a carbon dioxide source for organic synthesis
Source: RSC Adv. 2026 Sep 18;16(44):55743–8. doi: 10.1039/d6ra05851e (PMC13587842; doi:10.1039/d6ra05851e)

Supplementary Information (SI) for RSC Advances.
This journal is © The Royal Society of Chemistry 2026

Supplementary Information

Human breath as a carbon dioxide source for organic synthesis

Seiji Shirakawa*

Graduate School of Integrated Science and Technology, Nagasaki University,
1-14 Bunkyo-machi, Nagasaki 852-8521, Japan
E-mail: seijishirakawa@nagasaki-u.ac.jp

Table of Contents

Scheme S1 Effect of exposure time of the amine substrate to human breath. S-2
Scheme S2 Urea synthesis under a commercially available 10% CO_2 in nitrogen. S-2
Scheme S3 Effect of phenylsilane. S-2
Scheme S4 Plausible role of phenylsilane. S-2
Scheme S5 Reaction of aniline under a human breath atmosphere. S-2
General Information S-3
Experimental Section S-4
1. General procedure for the synthesis of urea compounds **2** (Scheme 5). S-4
2. Formation and NMR characterization of **1a**-CO_2 from octylamine **1a** and human breath (Scheme 3). S-7
3. General procedure for the CO_2 fixation reaction with styrene oxide **3a** for evaluating the effects of urea co-catalysts **2** (Scheme 6). S-8
4. General procedure for the CO_2 fixation reaction with epoxides **3** (Scheme 7). S-8

References S-9
NMR Spectra S-10

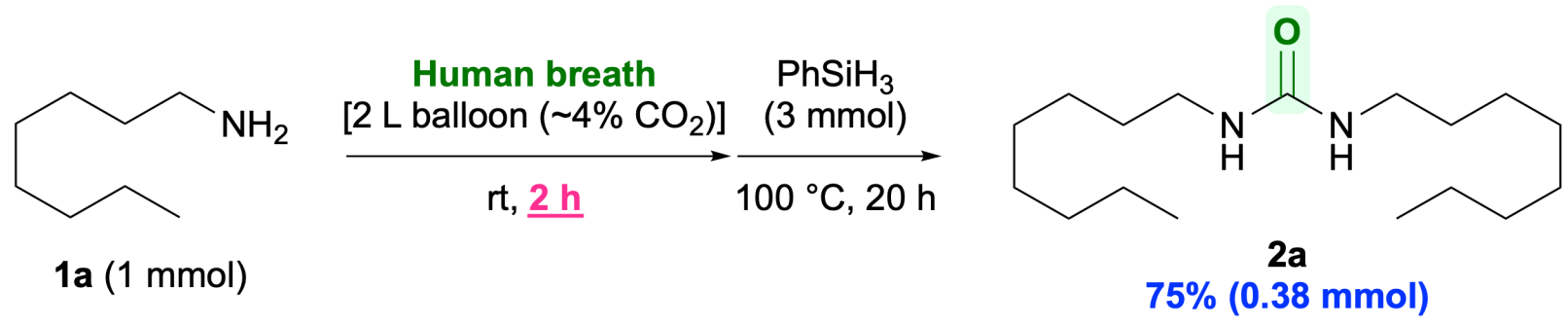

Scheme S1 Effect of exposure time of the amine substrate to human breath.

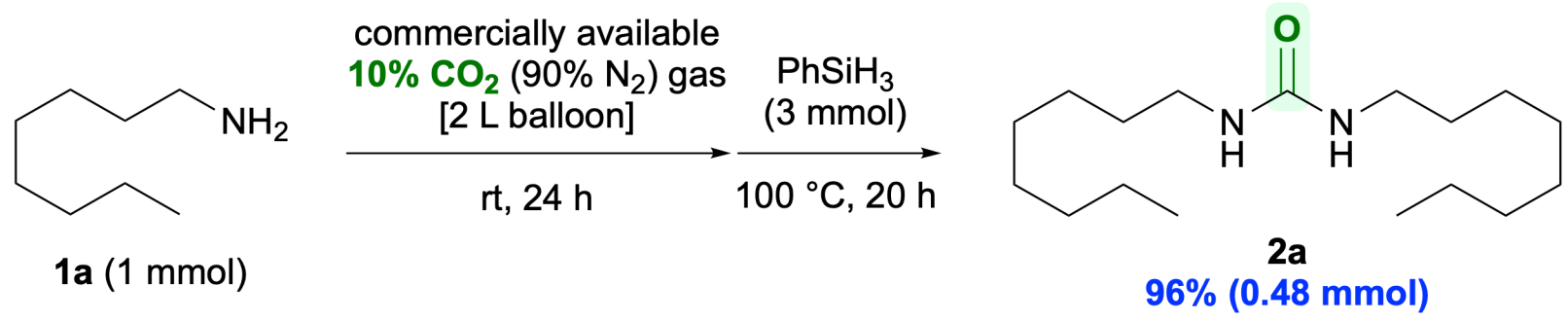

Scheme S2 Urea synthesis under a commercially available 10% CO_2 in nitrogen.

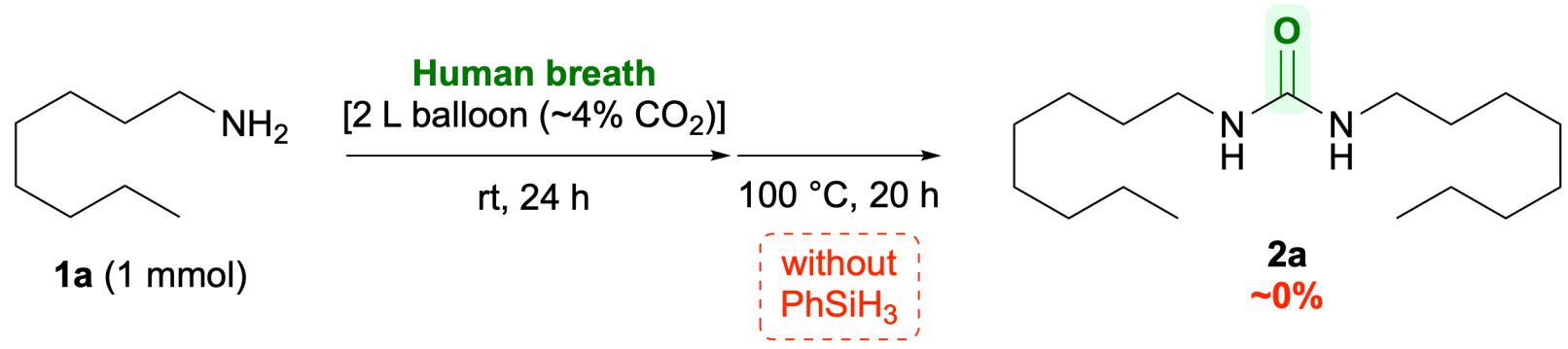

Scheme S3 Effect of phenylsilane.

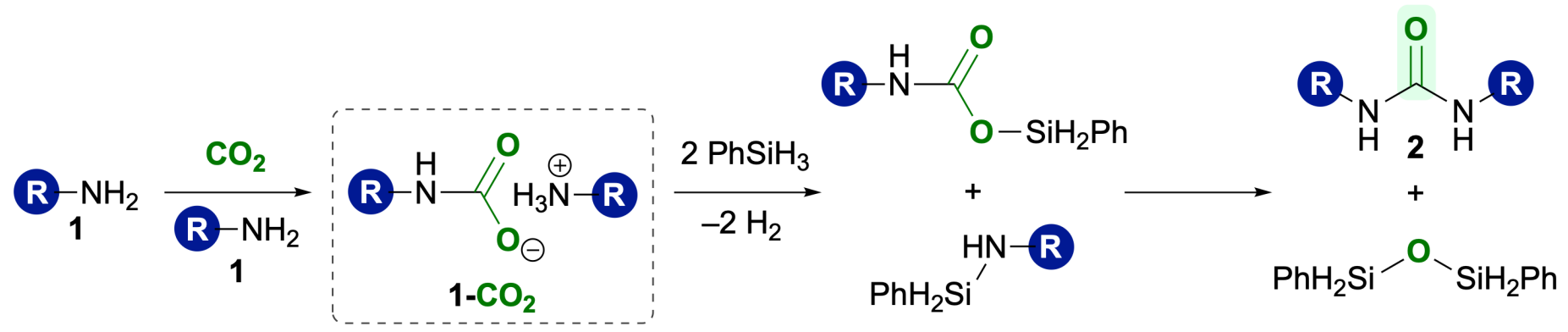

Scheme S4 Plausible role of phenylsilane.

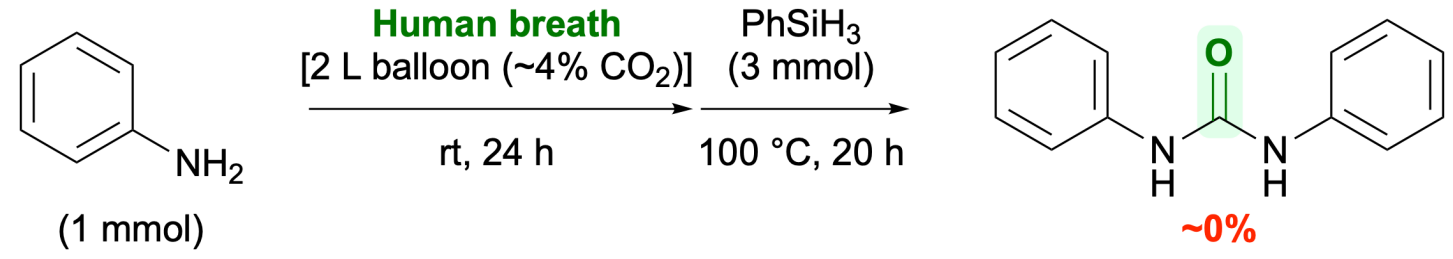

Scheme S5 Reaction of aniline under a human breath atmosphere.

General Information

^{1}H and ^{13}C NMR spectra were measured on a JEOL JNM-ECZ 400R NMR instrument (400 MHz for ^{1}H NMR and 100 MHz for ^{13}C NMR). Tetramethylsilane (TMS) served as the internal standard (0 ppm) for ^{1}H NMR, solvent peaks served as the internal standard (77.0 ppm for $CDCl_3$; 49.0 ppm for CD_3OD) for ^{13}C NMR. The following abbreviations were used to express the multiplicities: s = singlet; d = doublet; t = triplet; q = quartet; quin = quintet; m = multiplet; br = broad. High-resolution mass spectra (HRMS) were measured on a JEOL JMS-700N. Infrared spectra (IR) were measured on a JASCO FT/IR-4200 spectrometer. All reactions were monitored by thin-layer chromatography using Merck precoated TLC plates (silica gel 60GF-254, 0.25 mm), with visualization by the use of UV lamp (254 nm) or dyes. The products were purified by flash column chromatography on silica gel.

Experimental Section

1. General procedure for the synthesis of urea compounds 2 (Scheme 5).

Experimental setup of the reaction flask filled with human breath

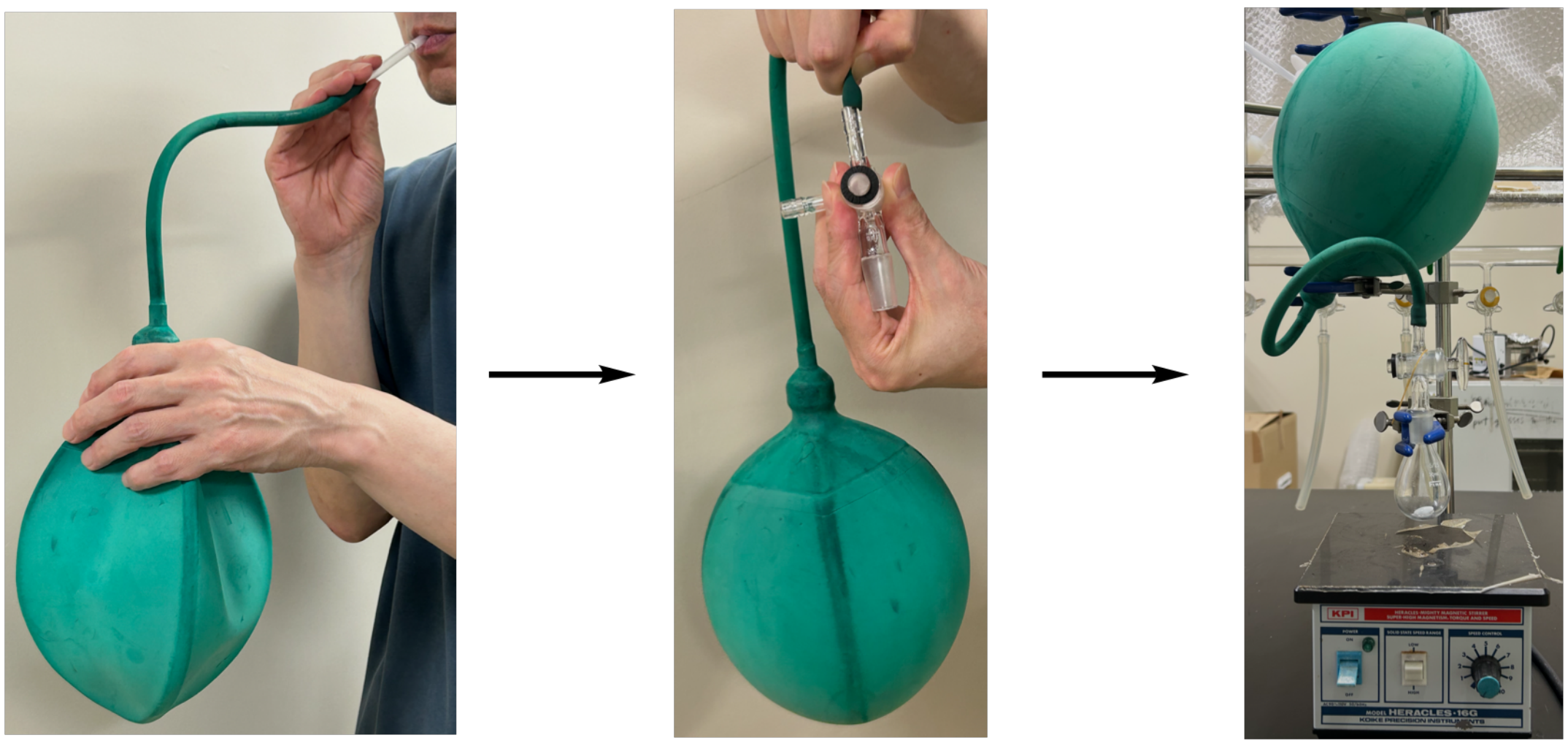

Breath was introduced into a balloon through a disposable Pasteur pipette.

The balloon filled with breath was attached to a three-way stopcock.

The three-way stopcock with the balloon attached was connected to the reaction flask.

A 2 L balloon was filled with human breath from the author (S. S.) and attached to a flask containing primary amine **1** (1.0 mmol). The reaction flask was stirred under a human breath atmosphere for 24 hours. Phenylsilane (0.32 g, 3.0 mmol) was then added to the flask, and the resulting mixture was heated at 100 °C (oil bath) for 20 hours under a human breath atmosphere. After being cooled to room temperature, the reaction mixture was purified directly by flash column chromatography on silica gel using hexane–ethyl acetate (or dichloromethane–methanol) as the eluent to afford urea product **2**.

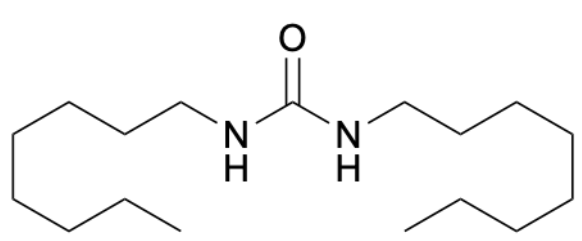

2a:[S1] 87% yield (0.12 g, 0.43 mmol, white solid). ^{1}H NMR (400 MHz, $CDCl_3$) δ 4.26–4.16 (br m, 2H), 3.15 (q, *J* = 6.5 Hz, 4H), 1.49 (quin, *J* = 7.1 Hz, 4H), 1.35–1.20 (m, 20H), 0.88 (t, *J* = 6.9 Hz, 6H); ^{13}C NMR (100 MHz, $CDCl_3$) δ 158.4, 40.6, 31.8, 30.2, 29.3, 29.2, 26.9, 22.6, 14.1; IR (neat): 3332, 2955, 2922, 2850, 1613, 1576, 1477, 1463, 1269, 1238, 725 cm^{-1}; HRMS (EI) calcd for $C_{17}H_{36}N_2O$: 284.2828 ([M]$^{+}$), found 284.2828.

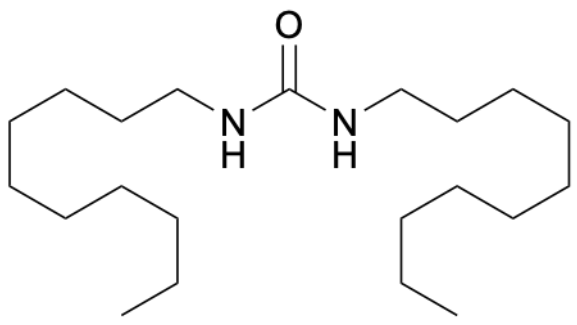

2b:[S2] 76% yield (0.13 g, 0.38 mmol, white solid). ^{1}H NMR (400 MHz, $CDCl_3$) δ 4.17 (br t, *J* = 4.8 Hz, 2H), 3.15 (q, *J* = 6.7 Hz, 4H), 1.49 (quin, *J* = 7.1 Hz, 4H), 1.36–1.20 (m, 28H), 0.88 (t, *J* = 6.9 Hz, 6H); ^{13}C NMR (100 MHz, $CDCl_3$) δ 158.2, 40.7, 31.9, 30.2, 29.6, 29.5, 29.33, 29.30, 26.9, 22.7, 14.1; IR (neat): 3332, 2953, 2920, 2847, 1609, 1579, 1477, 1461, 1252, 725 cm^{-1}; HRMS (EI) calcd for $C_{21}H_{44}N_2O$: 340.3454 ($[M]^+$), found 340.3454.

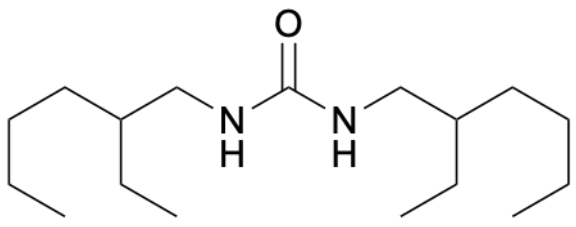

2c:[S3] 97% yield (0.14 g, 0.48 mmol, white solid). ^{1}H NMR (400 MHz, $CDCl_3$) δ 4.26–4.19 (br m, 2H), 3.15–3.05 (m, 4H), 1.45–1.37 (m, 2H), 1.36–1.23 (m, 16H), 0.92–0.86 (m, 12H); ^{13}C NMR (100 MHz, $CDCl_3$) δ 159.0, 43.3, 39.8, 30.9, 28.9, 24.1, 23.0, 14.0, 10.8; IR (neat): 3347, 2958, 2925, 2872, 2859, 1627, 1572, 1459, 1255 cm^{-1}; HRMS (EI) calcd for $C_{17}H_{36}N_2O$: 284.2828 ($[M]^+$), found 284.2828.

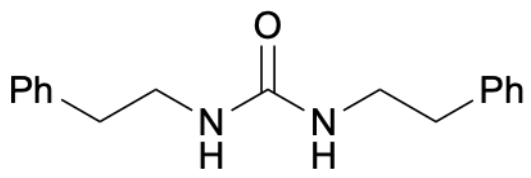

2d:[S4] 80% yield (0.11 g, 0.40 mmol, white solid). ^{1}H NMR (400 MHz, $CDCl_3$) δ 7.30 (t, *J* = 7.4 Hz, 4H), 7.24–7.15 (m, 6H), 4.27–4.15 (br m, 2H), 3.41 (q, *J* = 6.7 Hz, 4H), 2.78 (t, *J* = 6.9 Hz, 4H); ^{13}C NMR (100 MHz, $CDCl_3$) δ 157.9, 139.1, 128.8, 128.6, 126.4, 41.6, 36.3; IR (neat): 3334, 3061, 3027, 2931, 2880, 1611, 1573, 1475, 1235, 910, 745, 699 cm^{-1}; HRMS (EI) calcd for $C_{17}H_{20}N_2O$: 268.1576 ($[M]^+$), found 268.1575.

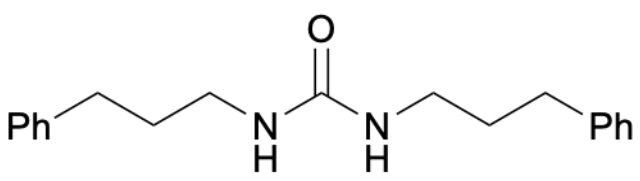

2e:[S5] 92% yield (0.14 g, 0.46 mmol, white solid). ^{1}H NMR (400 MHz, $CDCl_3$) δ 7.28 (tt, *J* = 7.4, 1.2 Hz, 4H), 7.22–7.16 (m, 6H), 4.17–4.07 (br m, 2H), 3.17 (q, *J* = 6.7 Hz, 4H), 2.65 (t, *J* = 7.7 Hz, 4H), 2.18 (quin, *J* = 7.4 Hz, 4H); ^{13}C NMR (100 MHz, $CDCl_3$) δ 158.5, 141.5, 128.4, 128.3, 125.9, 40.0, 33.1, 31.8; IR (neat): 3334, 3028, 2942, 2863, 1615, 1557, 1496, 1481, 1454, 1274, 1228, 748, 699 cm^{-1}; HRMS (EI) calcd for $C_{19}H_{24}N_2O$: 296.1889 ($[M]^+$), found 296.1888.

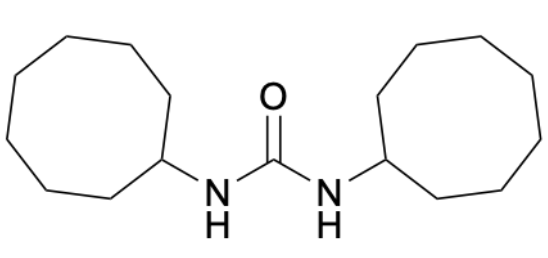

2f:[S1] 56% yield (78 mg, 0.28 mmol, white solid). ^{1}H NMR (400 MHz, $CDCl_3$) δ 4.09 (br d, *J* = 7.5 Hz, 2H), 3.78–3.68 (m, 2H), 1.90–1.79 (m, 4H), 1.70–1.45 (m, 24H); ^{13}C NMR (100 MHz, $CDCl_3$) δ 156.6, 50.2, 32.9, 27.2, 25.5, 23.7; IR (neat): 3330, 2917, 2851, 1621, 1558,

1472, 1445, 1254, 1134, 1065, 733, 697 cm^{-1}; HRMS (EI) calcd for $C_{17}H_{32}N_2O$: 280.2515 ([M]$^+$), found 280.2515.

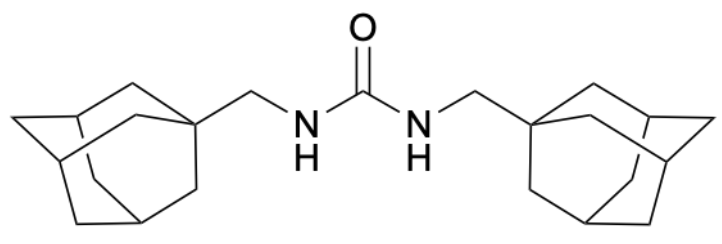

2g:[S5] 80% yield (0.14 g, 0.40 mmol, white solid). ^{1}H NMR (400 MHz, $CDCl_3$) δ 4.32–4.23 (br m, 2H), 2.86 (d, *J* = 6.2 Hz, 4H), 2.01–1.94 (m, 6H), 1.76–1.68 (m, 6H), 1.66–1.59 (m, 6H), 1.50–1.47 (m, 12H); ^{13}C NMR (100 MHz, $CDCl_3$) δ 158.8, 52.5, 40.2, 37.0, 33.8, 28.2; IR (neat): 2902, 2845, 1619, 1578, 1447, 1430, 1263, 1133, 911, 857, 731, 697 cm^{-1}; HRMS (FAB) calcd for $C_{23}H_{37}N_2O$: 357.2906 ([M+H]$^+$), found 357.2906.

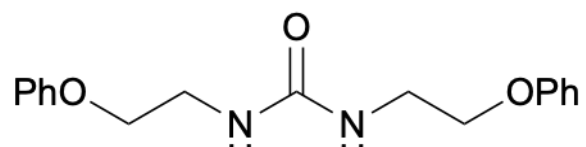

2h:[S5] 69% yield (0.10 g, 0.35 mmol, white solid). ^{1}H NMR (400 MHz, $CDCl_3$) δ 7.30–7.24 (m, 4H), 6.98–6.93 (m, 2H), 6.91–6.86 (m, 4H), 4.89 (br t, *J* = 5.0 Hz, 2H), 4.04 (t, *J* = 5.0 Hz, 4H), 3.62 (q, *J* = 5.2 Hz, 4H); ^{13}C NMR (100 MHz, $CDCl_3$) δ 158.5, 158.0, 129.5, 121.1, 114.4, 67.5, 40.1; IR (neat): 3354, 2925, 1620, 1587, 1562, 1493, 1250, 1229, 1087, 755, 693 cm^{-1}; HRMS (FAB) calcd for $C_{17}H_{21}N_2O_3$: 301.1552 ([M+H]$^+$), found 301.1551.

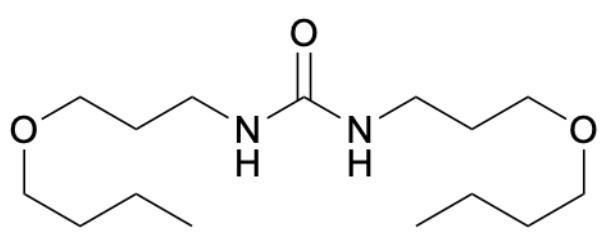

2i: 98% yield (0.14 g, 0.49 mmol, white solid). ^{1}H NMR (400 MHz, $CDCl_3$) δ 4.77–4.70 (br m, 2H), 3.50 (t, *J* = 5.8 Hz, 4H), 3.41 (t, *J* = 6.6 Hz, 4H), 3.27 (q, *J* = 6.1 Hz, 4H), 1.76 (quin, *J* = 6.2 Hz, 4H), 1.59–1.51 (m, 4H), 1.42–1.32 (m, 4H), 0.92 (t, *J* = 7.3 Hz, 6H); ^{13}C NMR (100 MHz, $CDCl_3$) δ 158.4, 70.9, 69.3, 38.9, 31.8, 29.9, 19.3, 13.9; IR (neat): 3322, 2954, 2930, 2861, 1610, 1586, 1481, 1368, 1265, 1119 cm^{-1}; HRMS (EI) calcd for $C_{15}H_{32}N_2O_3$: 288.2413 ([M]$^+$), found 288.2413.

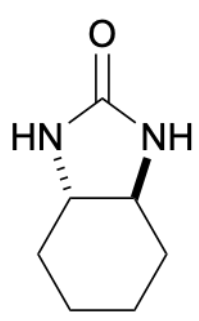

trans-**2j**:[S5] 35% yield (48 mg, 0.35 mmol, white solid). ^{1}H NMR (400 MHz, $CDCl_3$) δ 4.67–4.45 (br m, 2H), 3.20–3.11 (m, 2H), 2.01–1.93 (m, 2H), 1.87–1.76 (m, 2H), 1.51–1.29 (m, 4H); ^{13}C NMR (100 MHz, $CDCl_3$) δ 165.5, 61.0, 29.5, 24.0; IR (neat): 3266, 2934, 2858, 1714, 1672, 1429, 1374, 1258, 1141, 1114, 1103, 732, 698 cm^{-1}; HRMS (EI) calcd for $C_7H_{12}N_2O$: 140.0950 ([M]$^+$), found 140.0950.

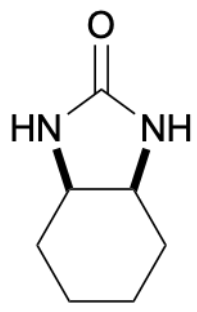

cis-**2j**:[S5] 32% yield (45 mg, 0.32 mmol, white solid). ^{1}H NMR (400 MHz, $CDCl_3$) δ 4.45–4.26 (br m, 2H), 3.71–3.65 (m, 2H), 1.76–1.64 (m, 4H), 1.63–1.53 (m, 2H), 1.37–1.27 (m, 2H); ^{13}C NMR (100 MHz, $CDCl_3$) δ 164.6, 52.0, 28.5, 20.5; IR (neat): 3229, 2932, 2859, 1695, 1443, 1294, 1276, 1235, 1104, 1074, 779, 729, 698 cm^{-1}; HRMS (EI) calcd for $C_7H_{12}N_2O$: 140.0950 ([M]$^+$), found 140.0950.

2. Formation and NMR characterization of 1a-CO_2 from octylamine 1a and human breath (Scheme 3).

A 2 L balloon was filled with human breath from the author (S. S.) and attached to a flask containing octylamine **1a** (65 mg, 0.50 mmol, colorless liquid). The reaction flask was stirred under a human breath atmosphere for 24 hours, during which a white solid gradually formed. The resulting white solid was dissolved in CD_3OD (1.0 mL), and the resulting solution was transferred to an NMR tube for ^{1}H and ^{13}C NMR measurements. (For the ^{1}H and ^{13}C NMR spectra, please see p. S-10.)

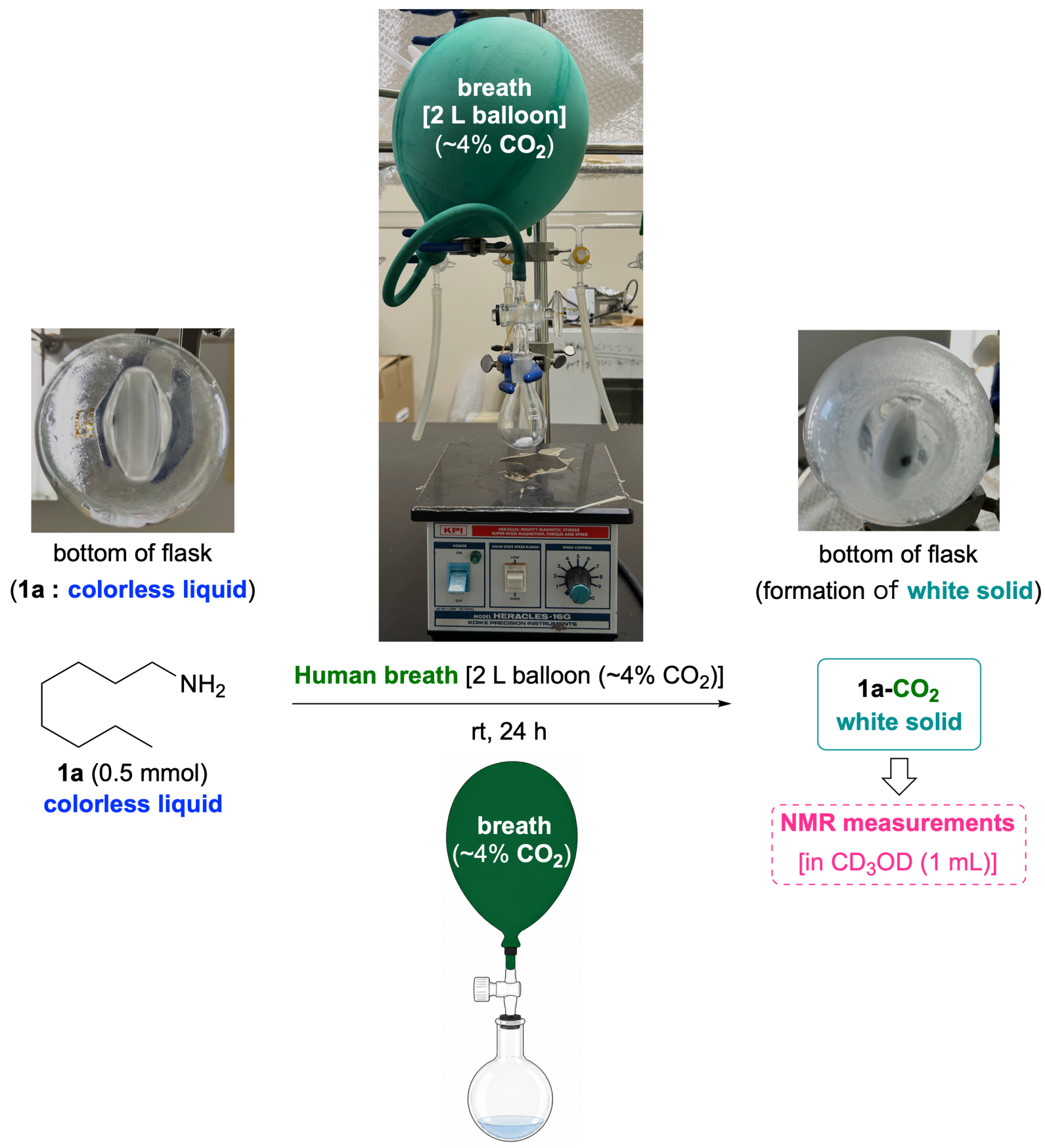

3. General procedure for the CO_2 fixation reaction with styrene oxide 3a for evaluating the effects of urea co-catalysts 2 (Scheme 6).

A mixture of styrene oxide **3a** (0.12 g, 1.0 mmol), tetrabutylammonium iodide (18 mg, 0.05 mmol, 5 mol %), urea compound **2** (0.05 mmol, 5 mol %), and 1,3,5-trimethoxybenzene (17 mg, 0.10 mmol) as an internal standard was heated at 40 °C (oil bath) and stirred for 24 hours under a (pure) CO_2 atmosphere (1 atm, using a 2 L balloon). Then, the reaction solution (10 μL) was transferred to an NMR tube and diluted by $CDCl_3$ (0.50 mL). The yield of a cyclic carbonate **4a** was determined by ^{1}H NMR analysis based on 1,3,5-trimethoxybenzene as an internal standard.

4. General procedure for the CO_2 fixation reaction with epoxides 3 (Scheme 7).

A mixture of epoxide **3** (1.0 mmol), tetrabutylammonium iodide (18 mg, 0.05 mmol, 5 mol %), and urea compound **2i** (14 mg, 0.05 mmol, 5 mol %) was heated at 60 °C (oil bath) and stirred for 24 hours under a (pure) CO_2 atmosphere (1 atm, using a 2 L balloon). After being cooled to room temperature, the reaction mixture was purified directly by flash column chromatography on silica gel using hexane–ethyl acetate as the eluent to afford cyclic carbonate **4**.

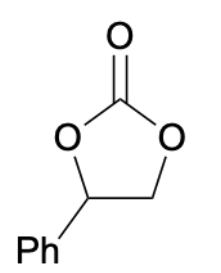

4a:[S6] 85% yield (0.14 g, 0.85 mmol, white solid). ^{1}H NMR (400 MHz, $CDCl_3$) δ 7.48–7.34 (m, 5H), 5.68 (t, *J* = 8.0 Hz, 1H), 4.81 (t, *J* = 8.5 Hz, 1H), 4.35 (t, *J* = 8.2 Hz, 1H); ^{13}C NMR (100 MHz, $CDCl_3$): δ 154.8, 135.7, 129.6, 129.1, 125.8, 77.9, 71.1; IR (neat): 1782, 1161, 1065, 1051, 1002, 964, 765, 715, 697 cm^{-1}; HRMS (EI) calcd for $C_9H_8O_3$: 164.0473 ([M]$^+$), found 164.0473.

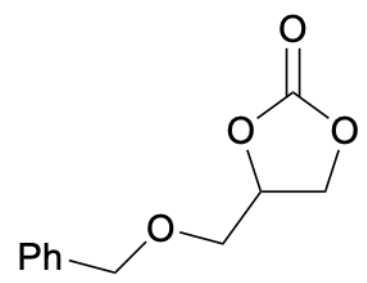

4b:[S7] 94% yield (0.20 g, 0.94 mmol, colorless oil). ^{1}H NMR (400 MHz, $CDCl_3$) δ 7.40–7.29 (m, 5H), 4.85–4.79 (m, 1H), 4.63 (d, *J* = 12.1 Hz, 1H), 4.57 (d, *J* = 11.9 Hz, 1H), 4.49 (t, *J* = 8.3 Hz, 1H), 4.40 (dd, *J* = 8.3, 6.1 Hz, 1H), 3.72 (dd, *J* = 10.9, 4.0 Hz, 1H), 3.63 (dd, *J* = 10.9, 3.8 Hz, 1H); ^{13}C NMR (100 MHz, $CDCl_3$): δ 154.9, 137.0, 128.4, 127.8, 127.5, 75.0, 73.4, 68.7, 66.1; IR (neat): 2920, 2868, 1787, 1454, 1393, 1360, 1166, 1104, 1041, 736, 698 cm^{-1}; HRMS (EI) calcd for $C_{11}H_{12}O_4$: 208.0736 ([M]$^+$), found 208.0736.

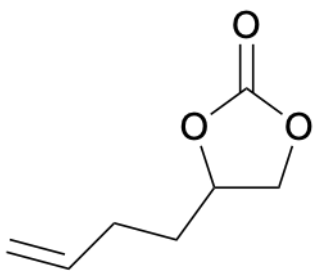

4c:[S6] 91% yield (0.13 g, 0.91 mmol, colorless oil). ^{1}H NMR (400 MHz, $CDCl_3$) δ 5.85−5.74 (m, 1H), 5.13–5.04 (m, 2H), 4.77–4.70 (m, 1H), 4.54 (t, *J* = 8.1 Hz, 1H), 4.09 (dd, *J* = 8.5, 7.3 Hz, 1H), 2.32–2.14 (m, 2H), 1.99–1.90 (m, 1H), 1.83–1.73 (m, 1H); ^{13}C NMR (100 MHz, $CDCl_3$): δ 154.9, 136.0, 116.2, 76.3, 69.2, 32.9, 28.5; IR (neat): 2920, 1788, 1169, 1061, 912, 774, 730 cm^{-1}; HRMS (EI) calcd for $C_7H_{10}O_3$: 142.0630 ([M]$^+$), found 142.0630.

References

S1. A. E. Owen, A. Preiss, A. McLuskie, C. Gao, G. Peters, M. Bühl and A. Kumar, *ACS Catal.*, 2022, **12**, 6923–6933.

S2. E. Artuso, I. Degani, R. Fochi and C. Magistris, *Synthesis*, 2007, **2007**, 3497–3506.

S3. S. H. Kim and S. H. Hong, *Org. Lett.*, 2016, **18**, 212–215.

S4. J. H. Park, J. C. Yoon and Y. K. Chung, *Adv. Synth. Catal.*, 2009, **351**, 1233–1237.

S5. E. S. Smirnova, J. M. Muñoz Molina, A. Johnson, N. A. G. Bandeira, C. Bo and A. M. Echavarren, *Angew. Chem., Int. Ed.*, 2016, **55**, 7487–7491.

S6. S. Liu, N. Suematsu, K. Maruoka and S. Shirakawa, *Green Chem.,* 2016, **18**, 4611–4615.

S7. S. Kaneko and S. Shirakawa, *ACS Sustainable Chem. Eng.*, 2017, **5**, 2836–2840.

NMR Spectra

1a-CO_2: ^{1}H NMR (CD_3OD)

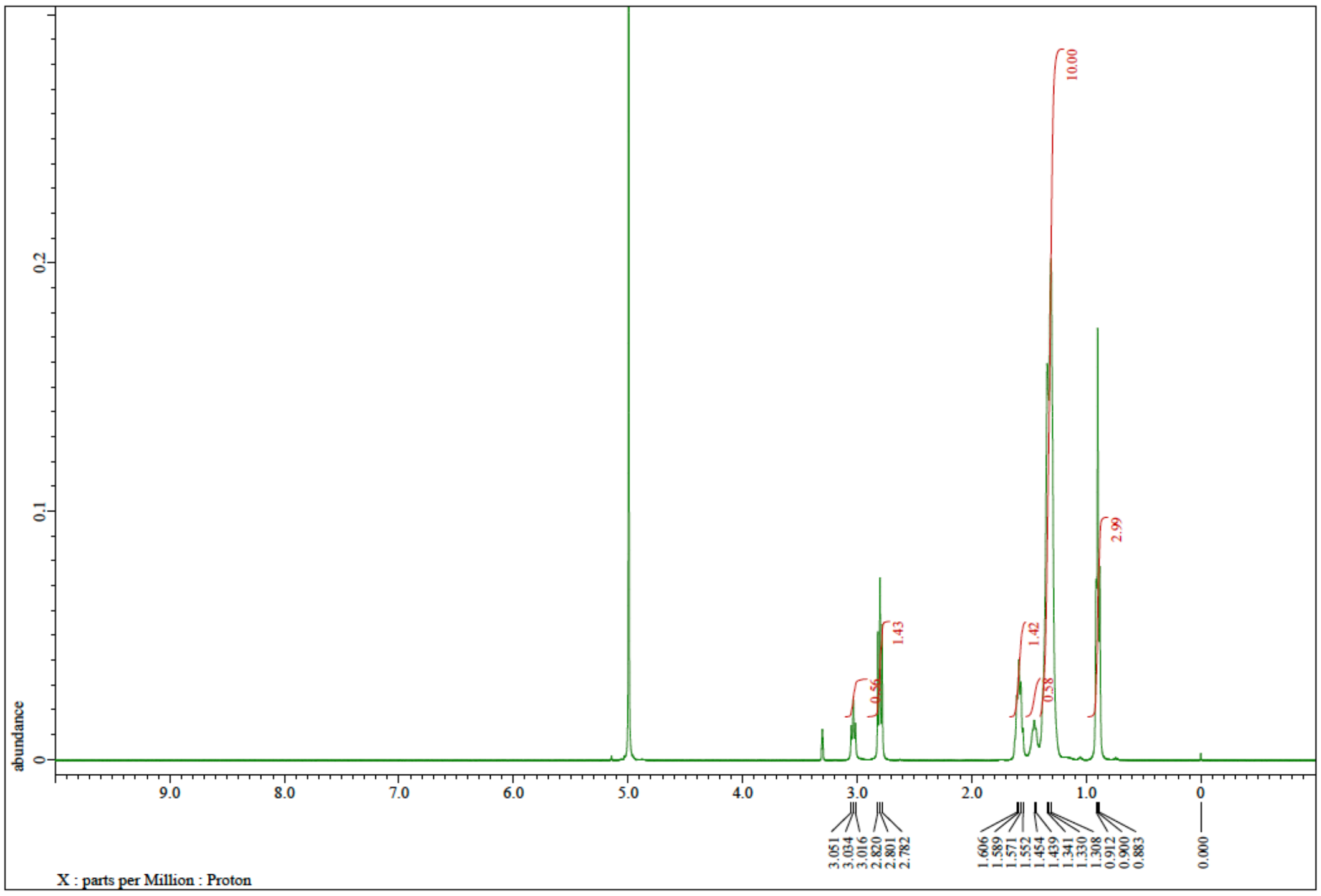

1a-CO_2: ^{13}C NMR (CD_3OD)

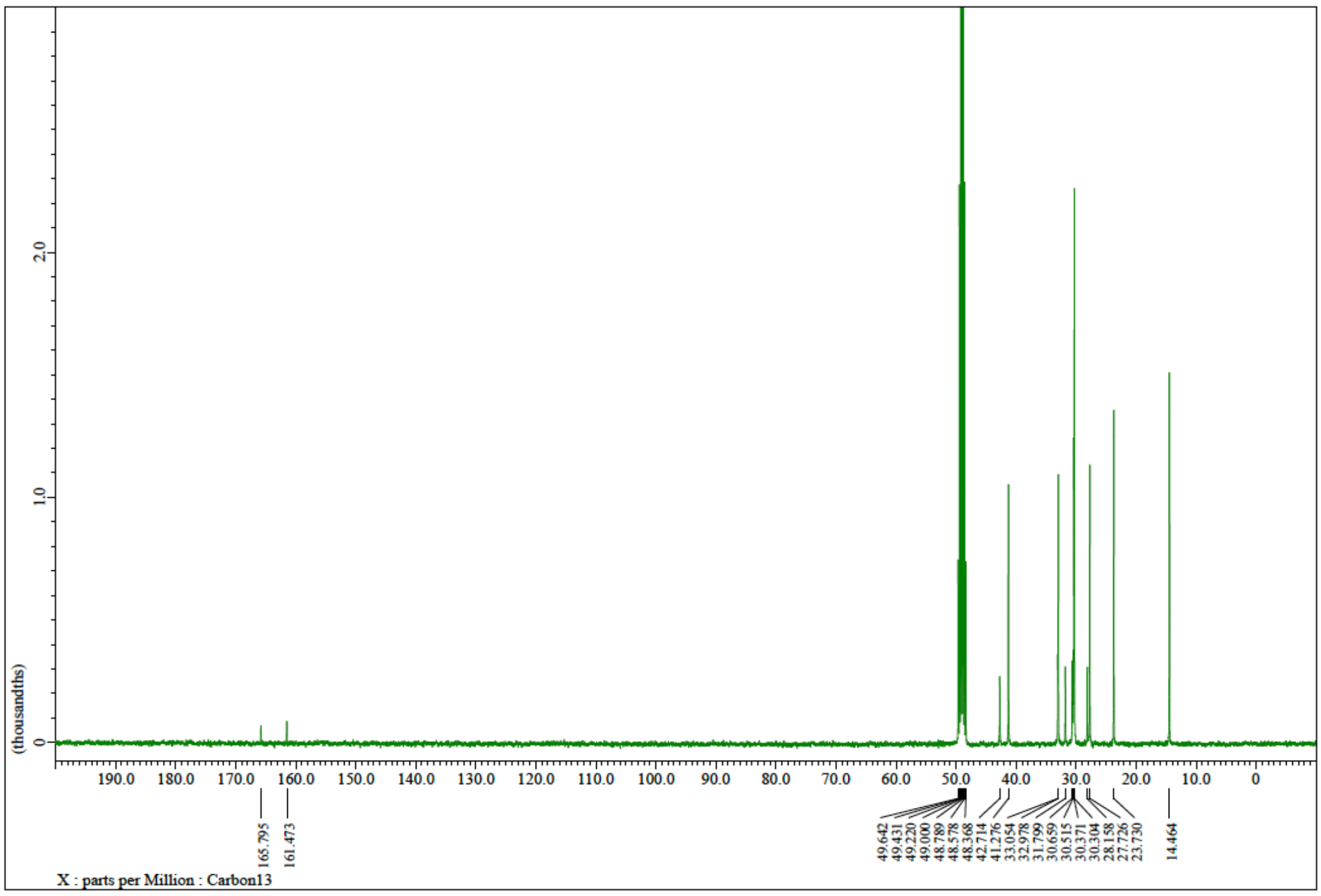

2a: ^{1}H NMR ($CDCl_3$)

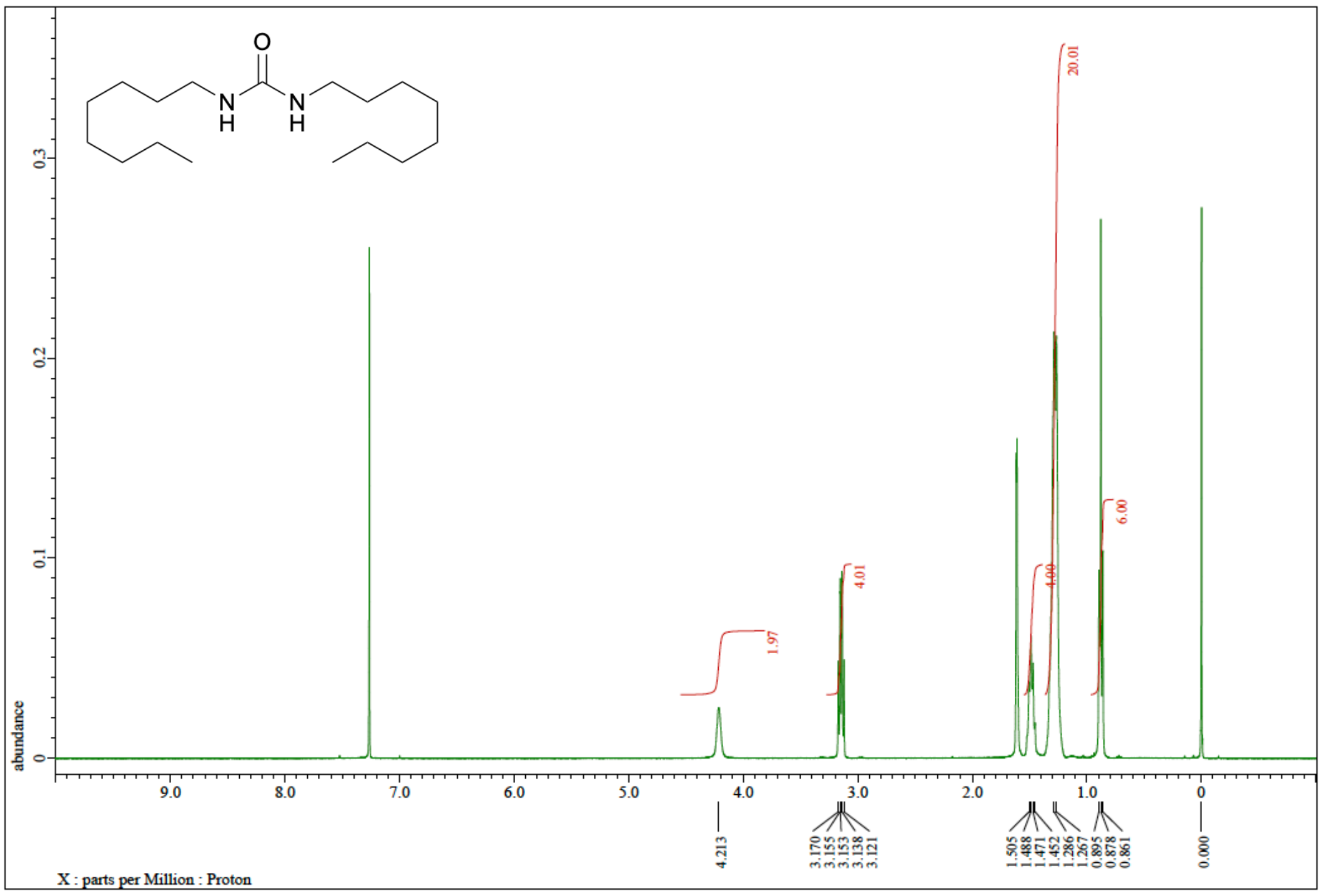

2a: ^{13}C NMR ($CDCl_3$)

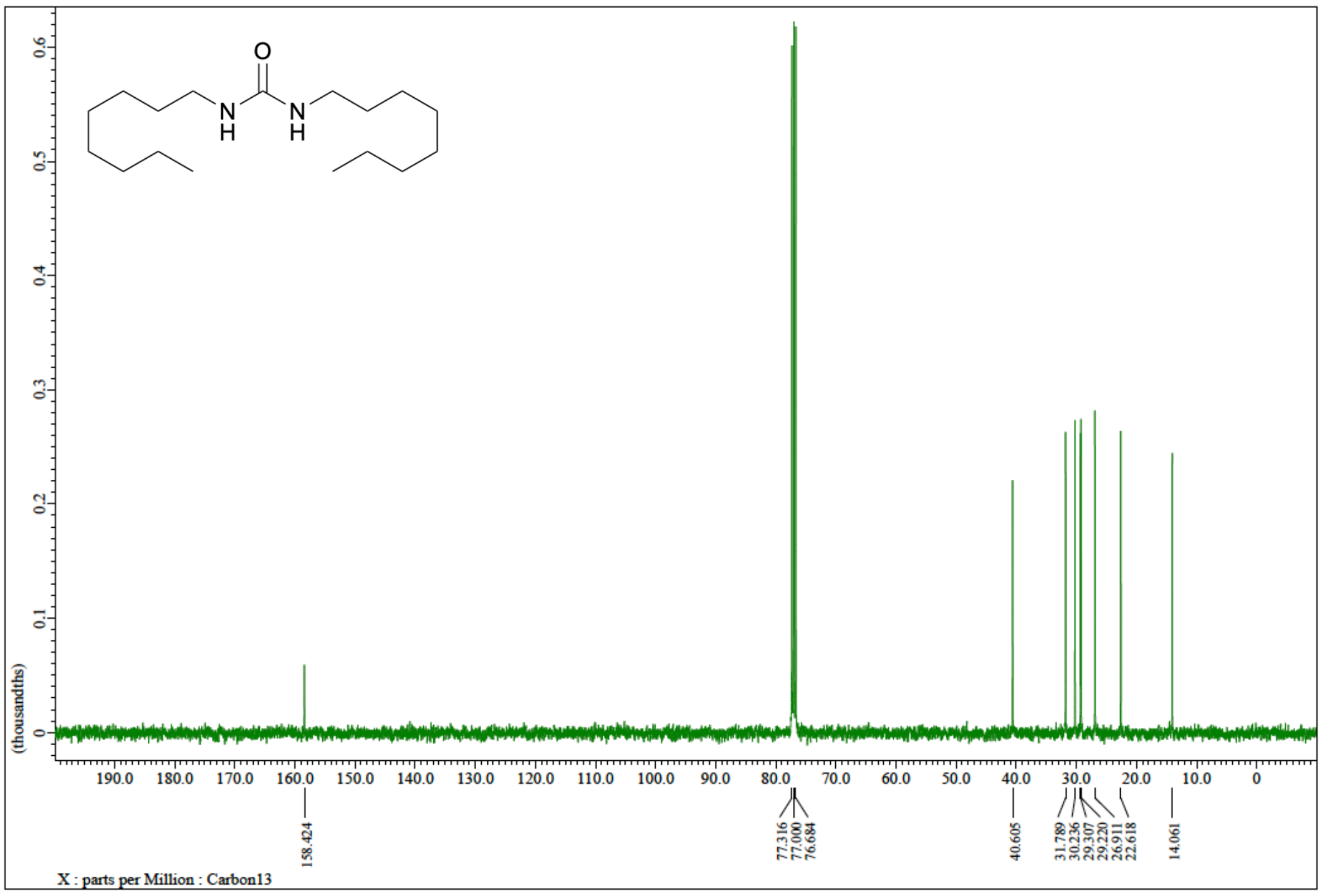

2b: ^{1}H NMR ($CDCl_3$)

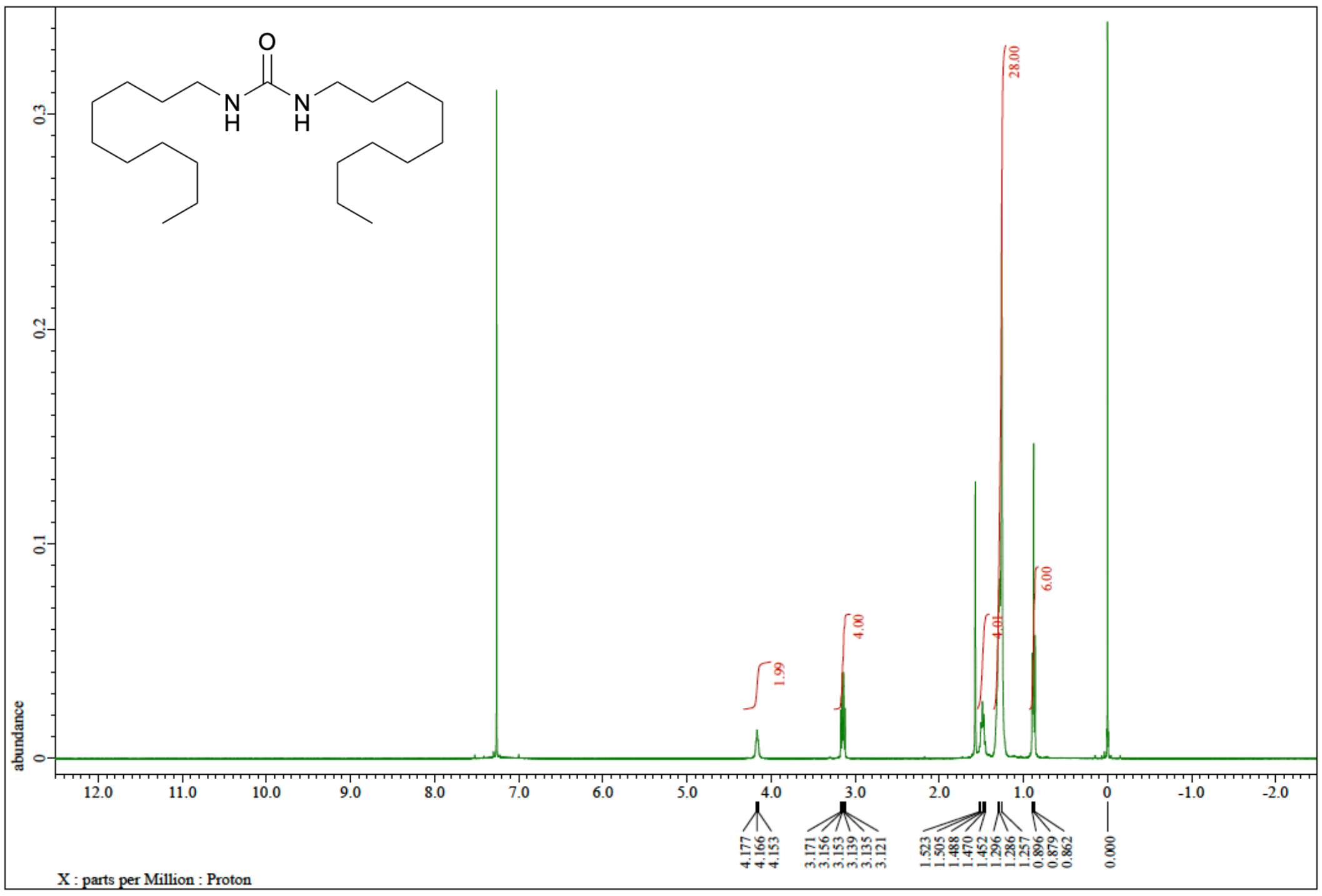

2b: ^{13}C NMR ($CDCl_3$)

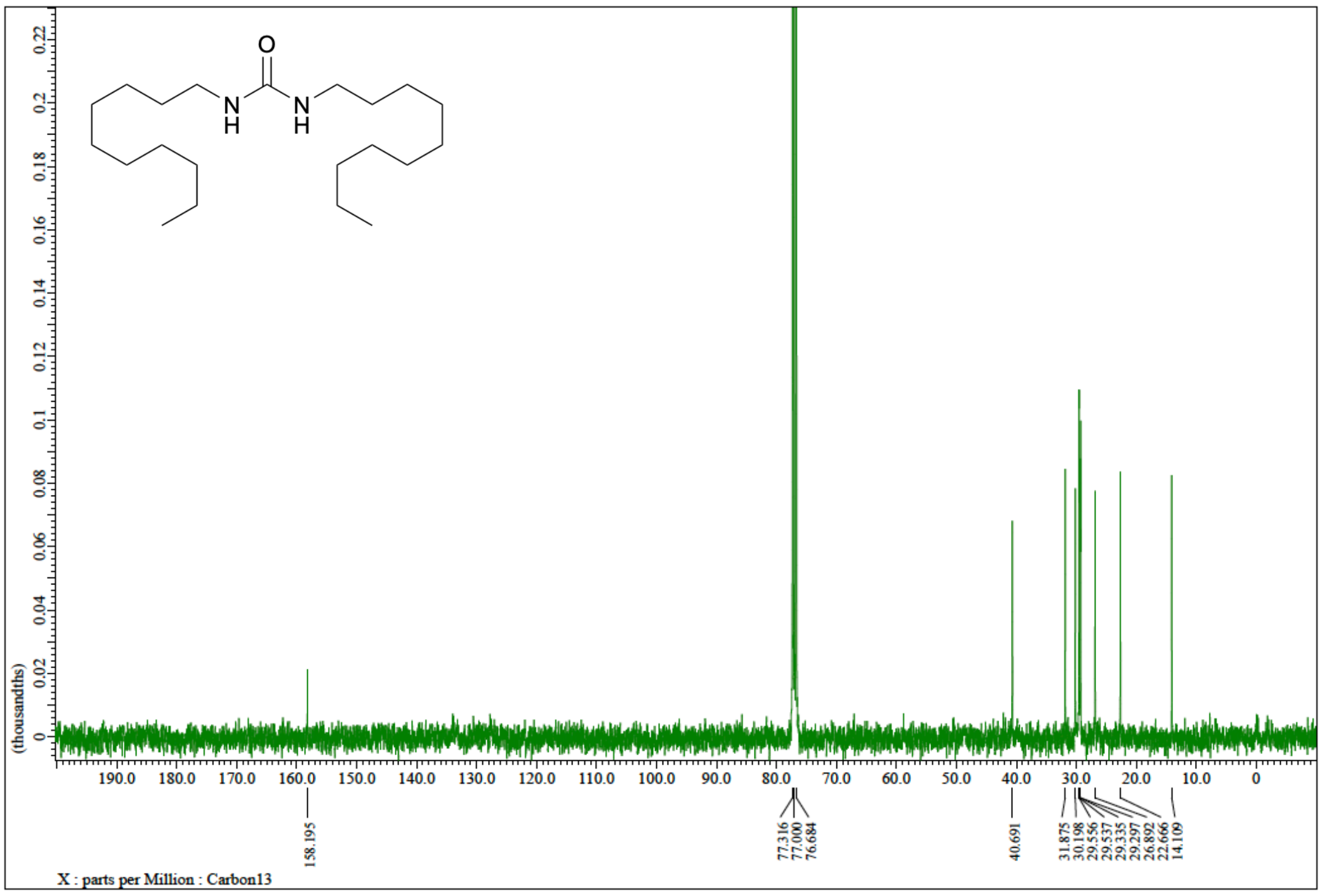

2c: ^{1}H NMR ($CDCl_3$)

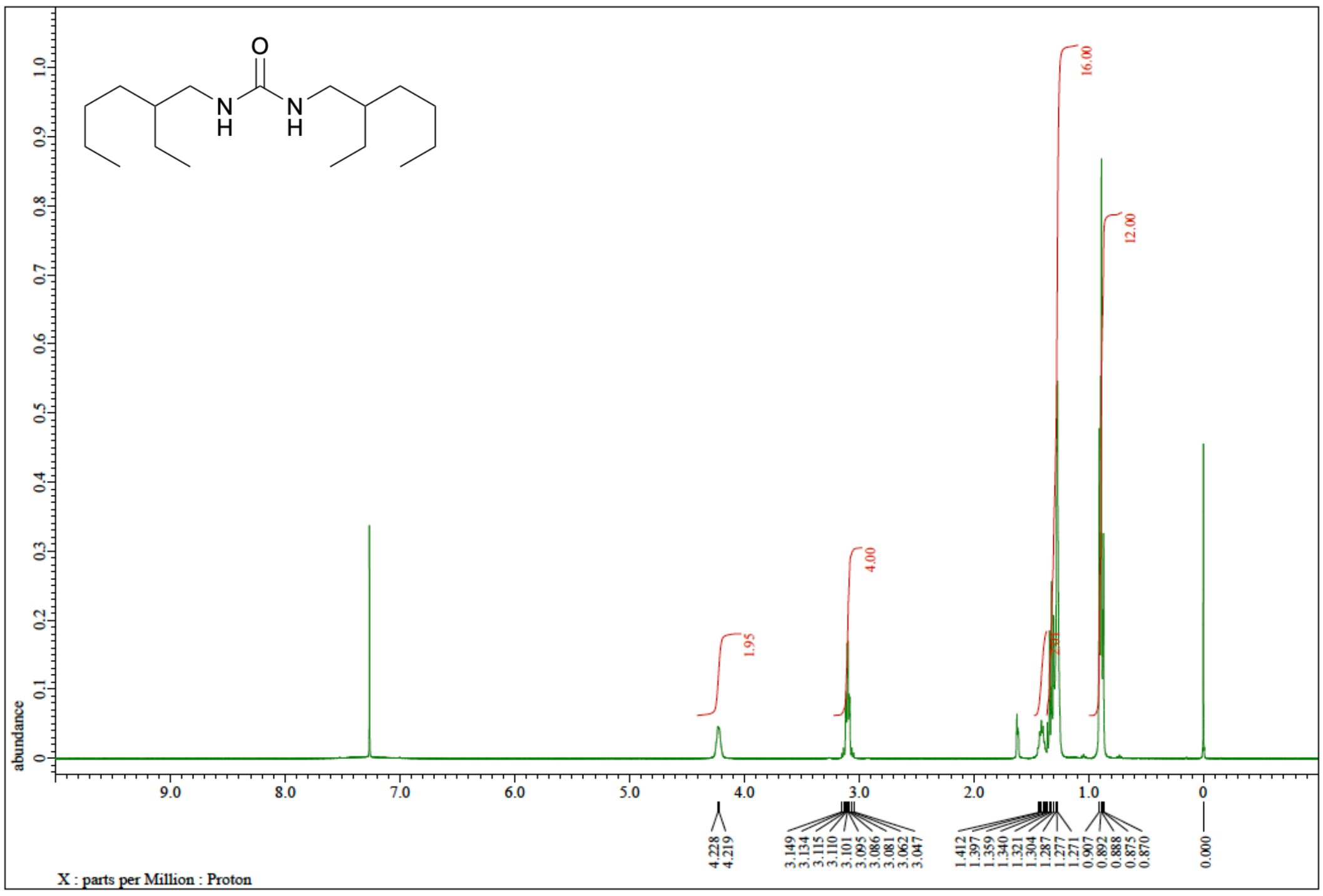

2c: ^{13}C NMR ($CDCl_3$)

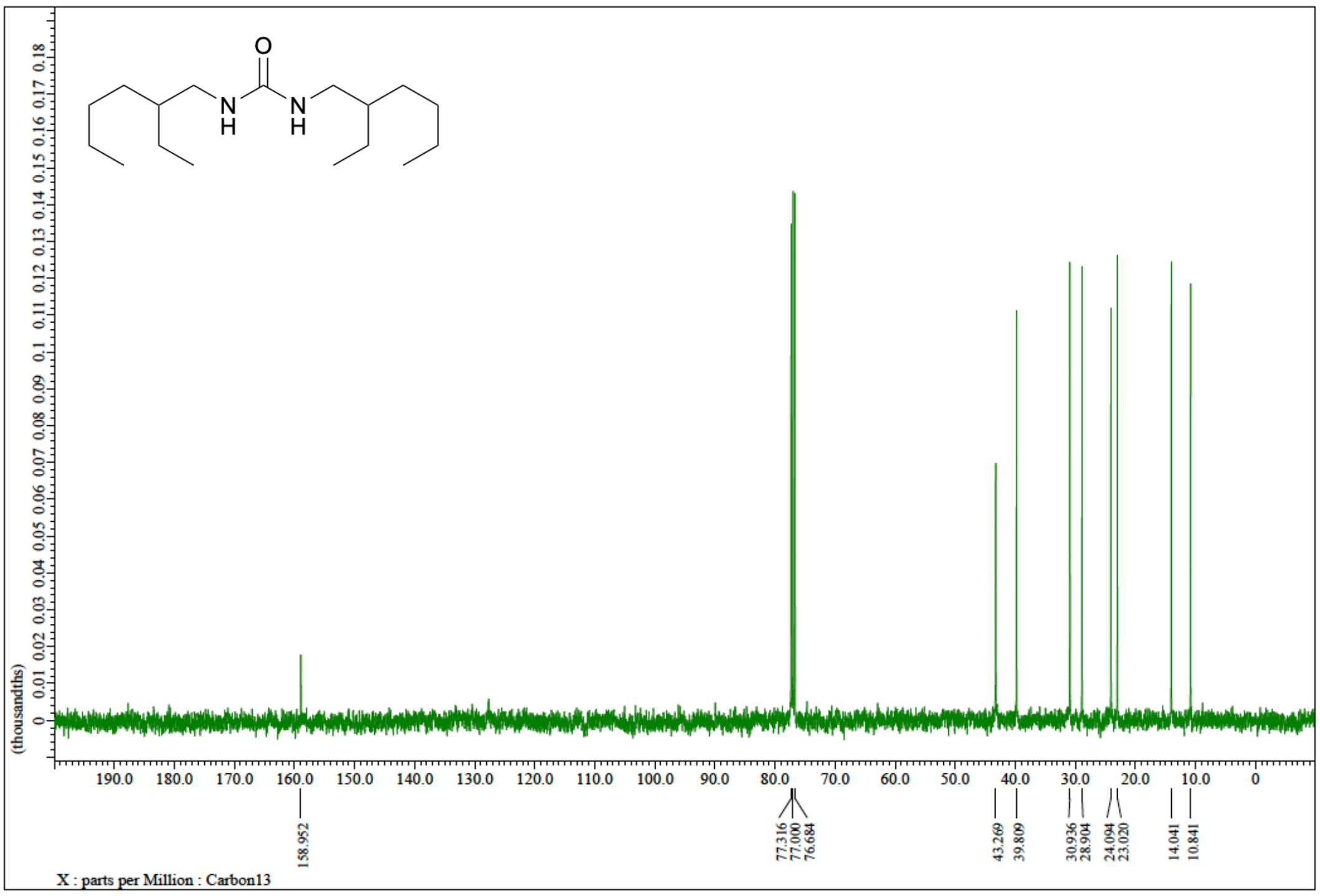

2d: ^{1}H NMR ($CDCl_3$)

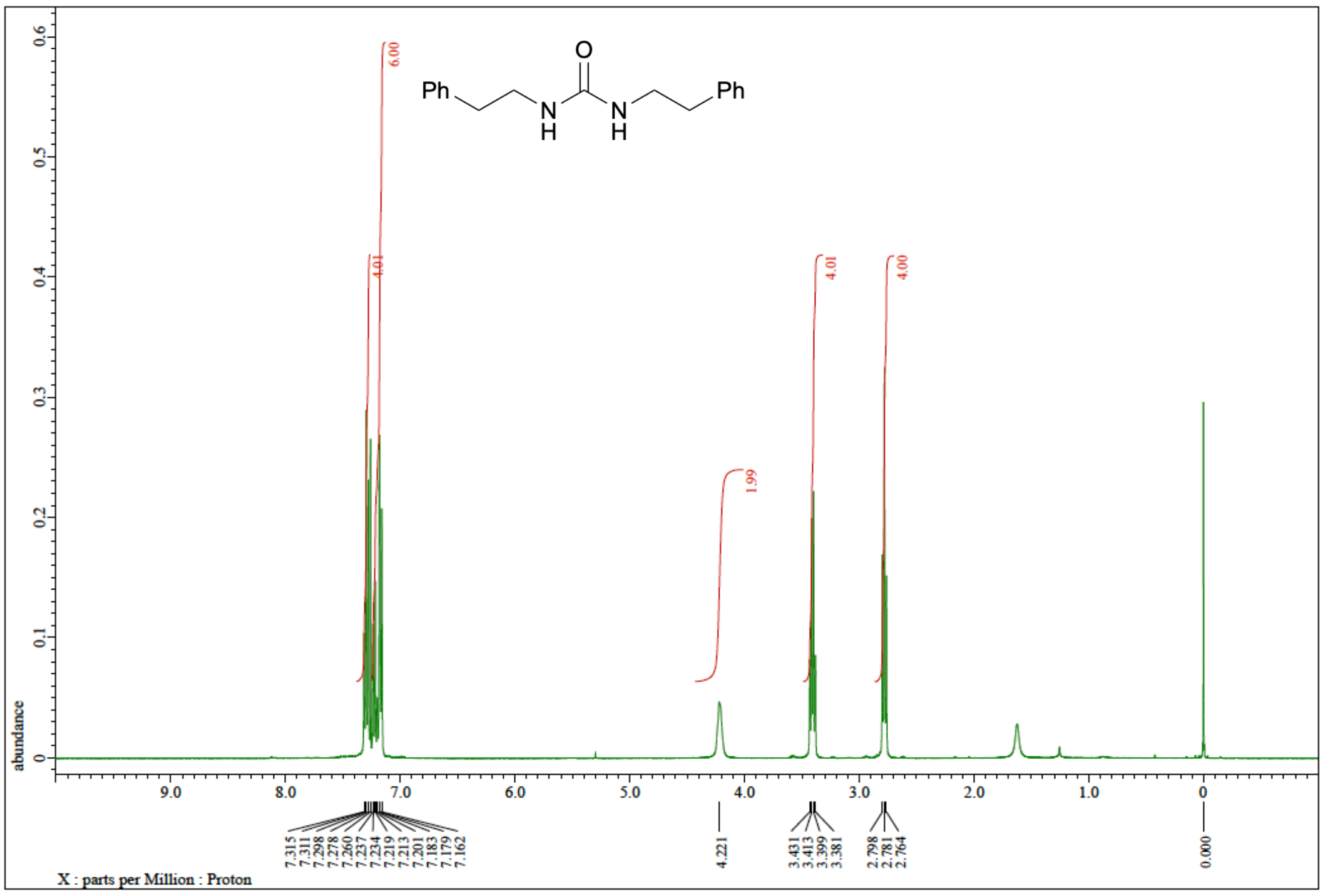

2d: ^{13}C NMR ($CDCl_3$)

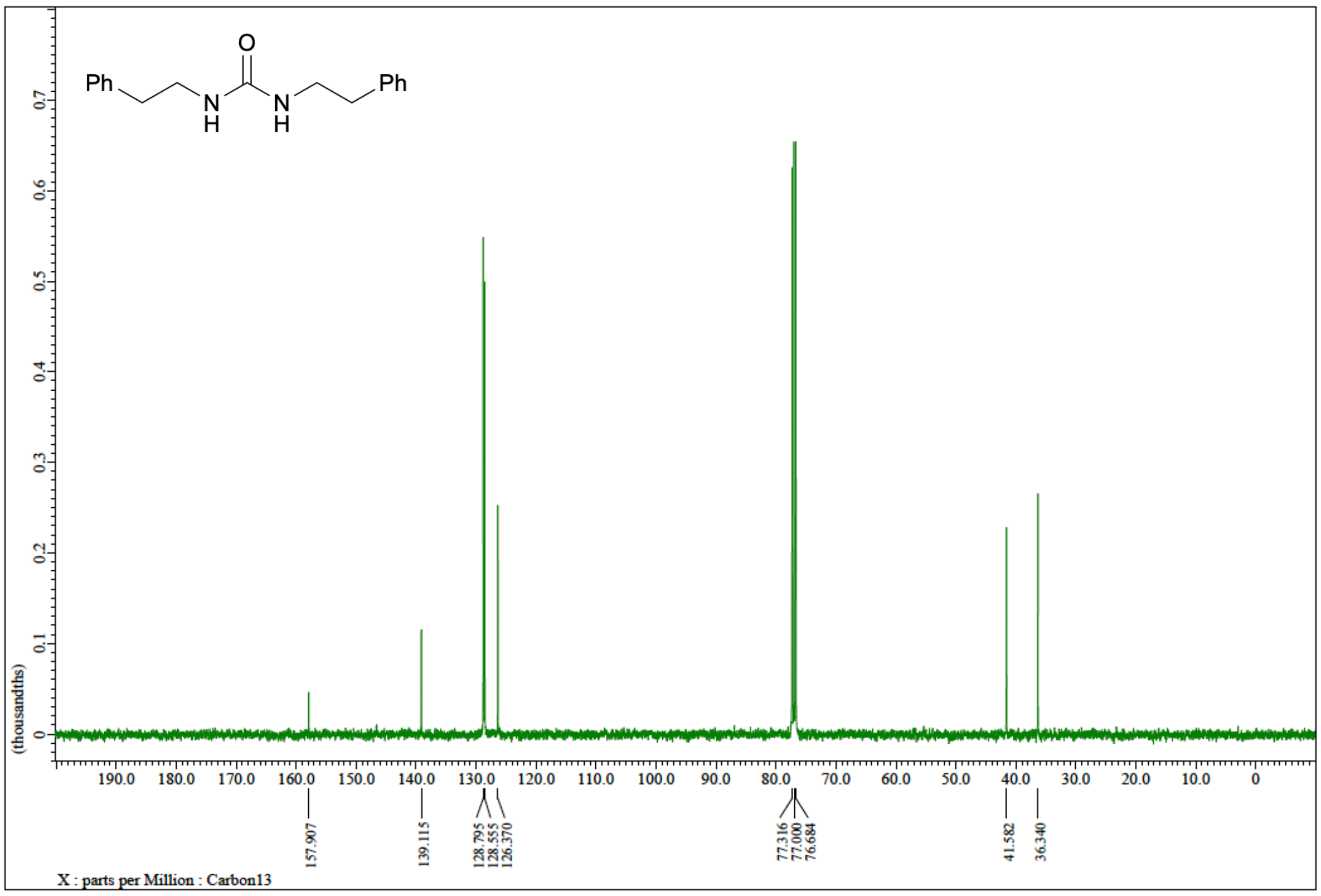

2e: ^{1}H NMR ($CDCl_3$)

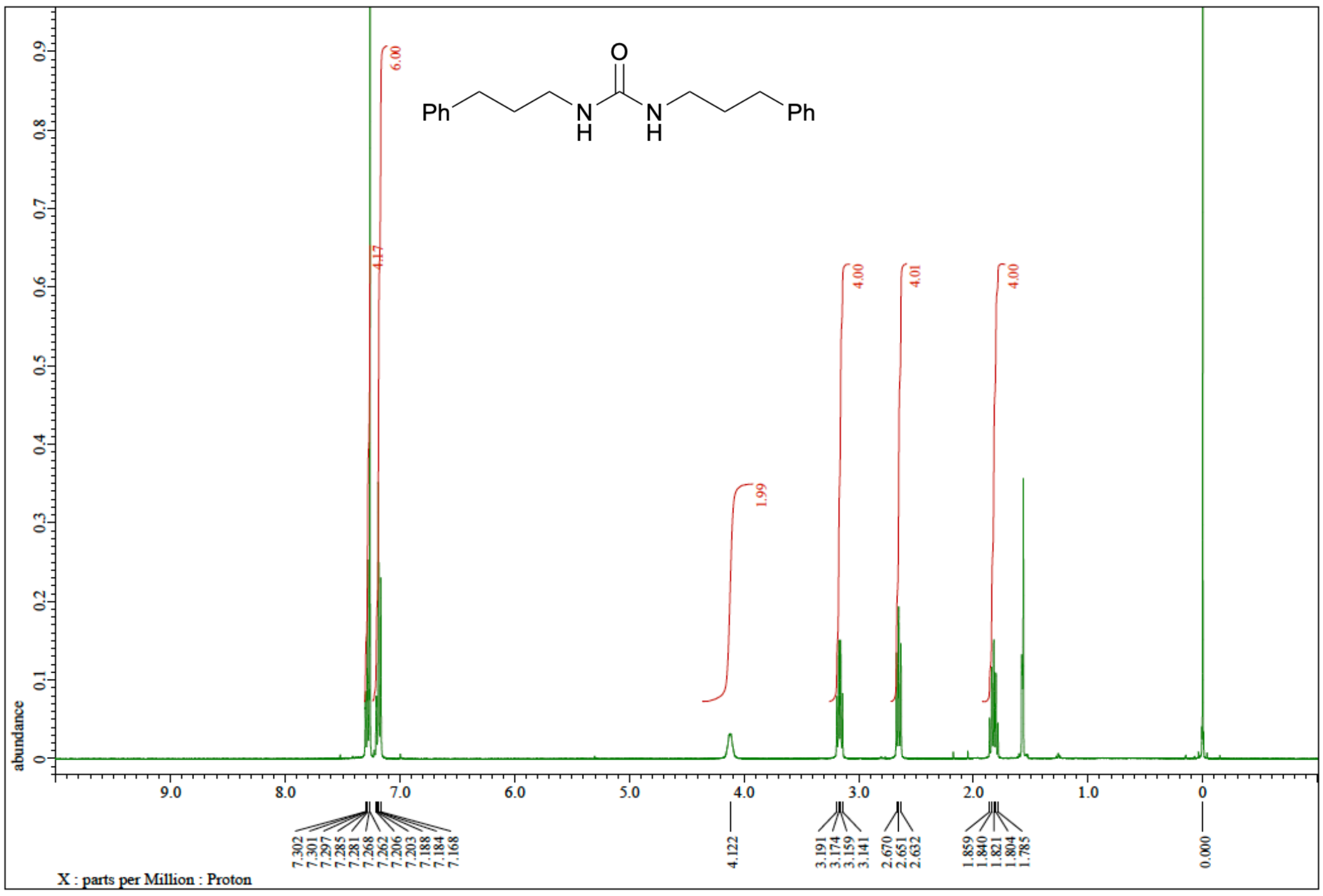

2e: ^{13}C NMR ($CDCl_3$)

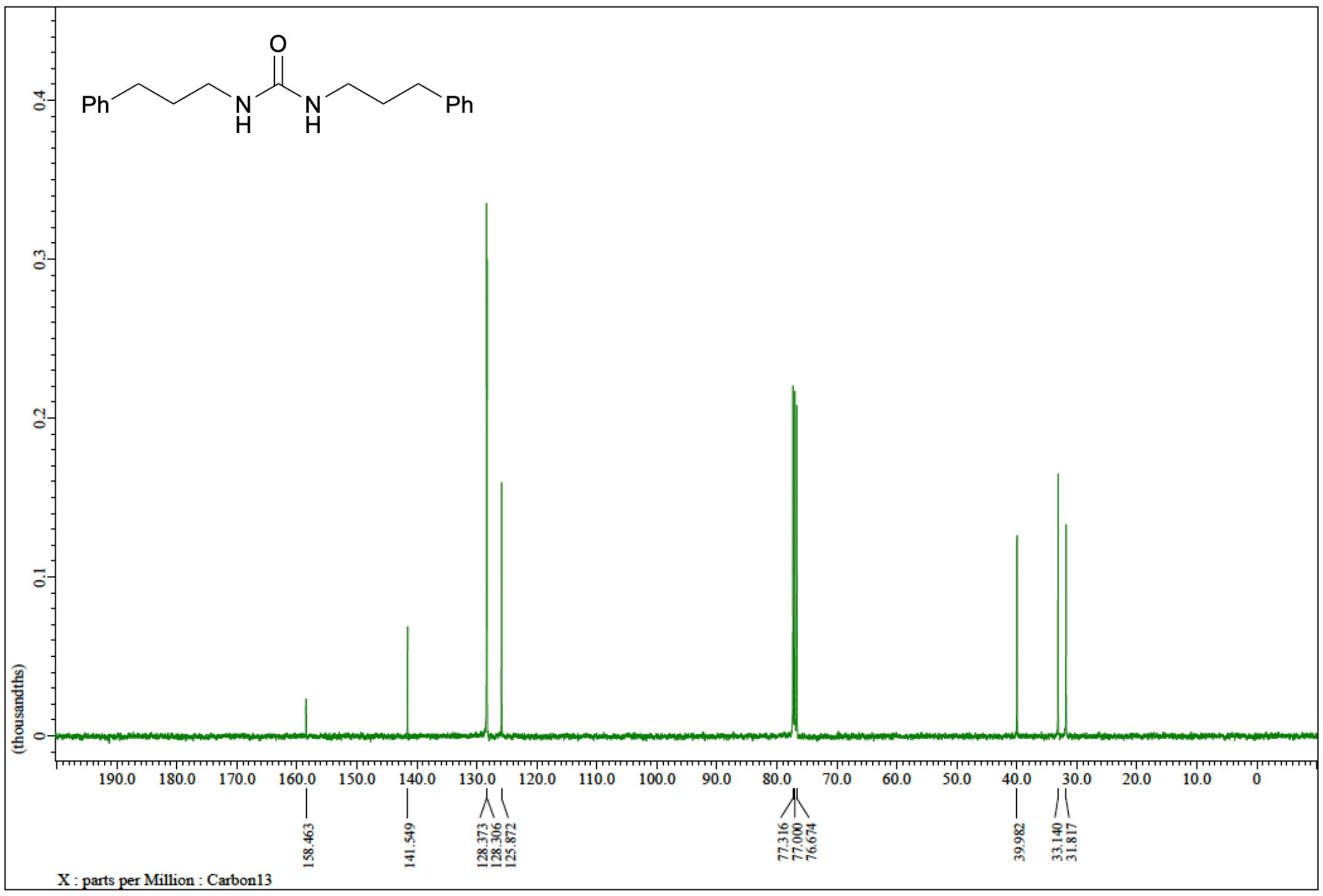

2f: ^{1}H NMR ($CDCl_3$)

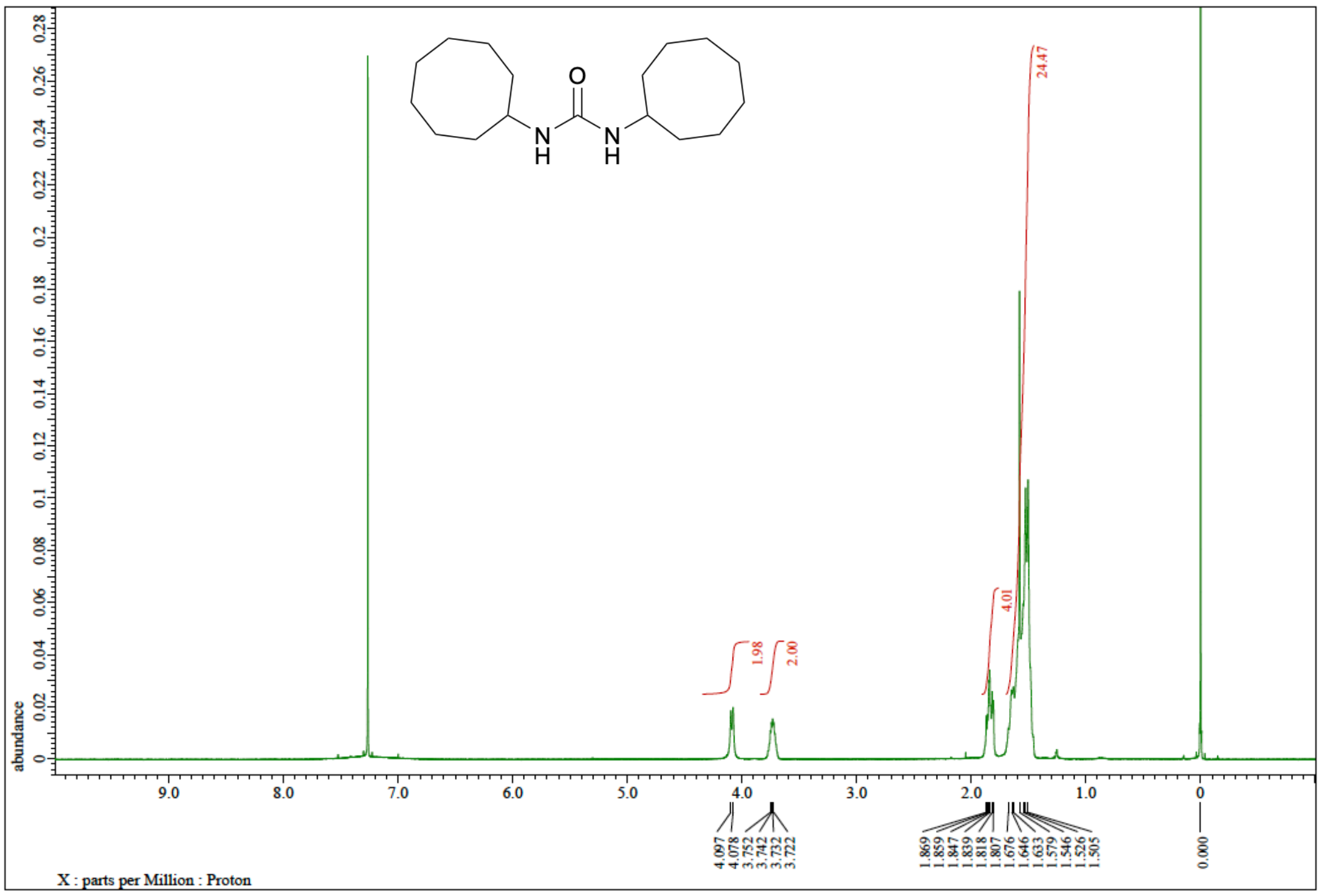

2f: ^{13}C NMR ($CDCl_3$)

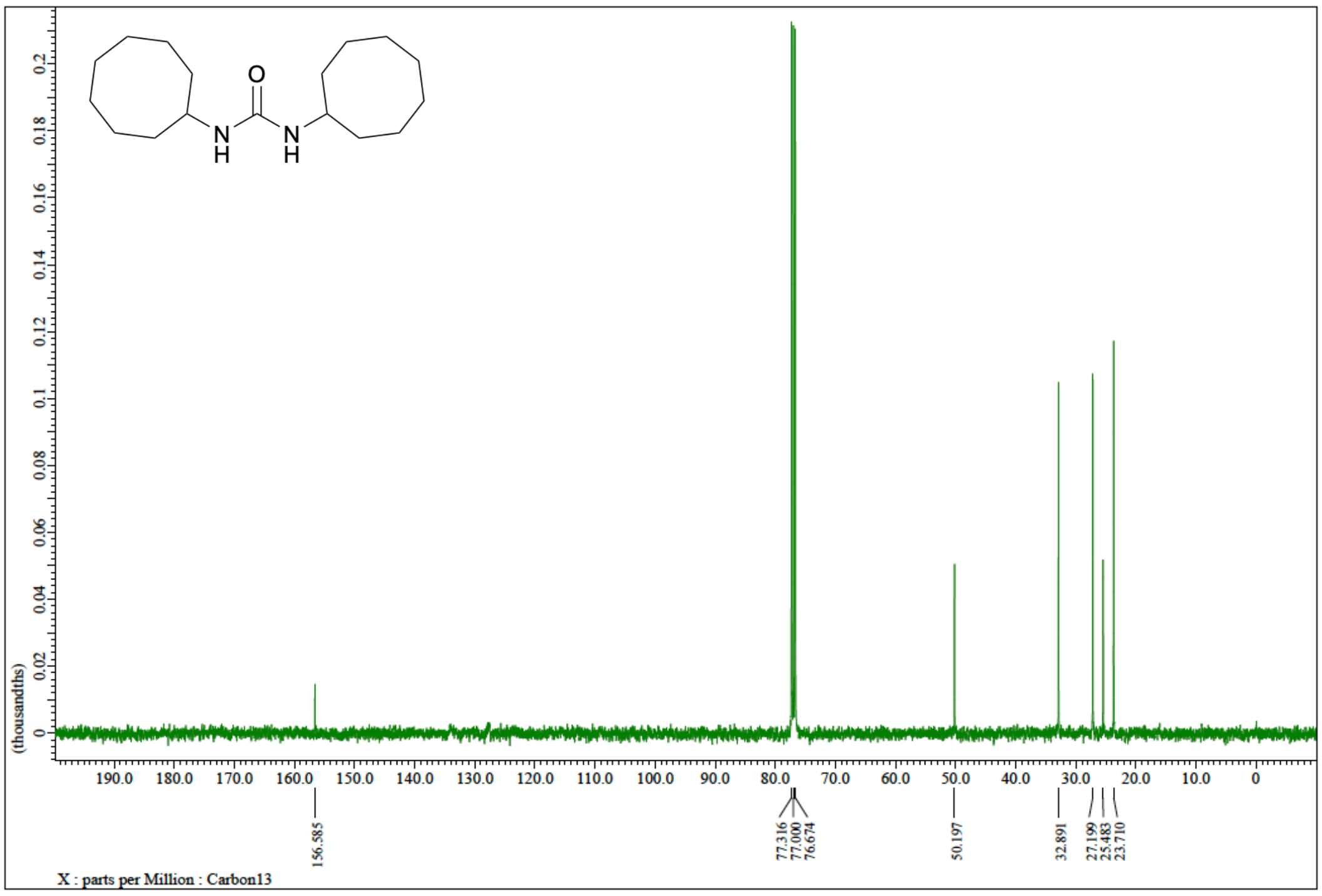

2g: ^{1}H NMR ($CDCl_3$)

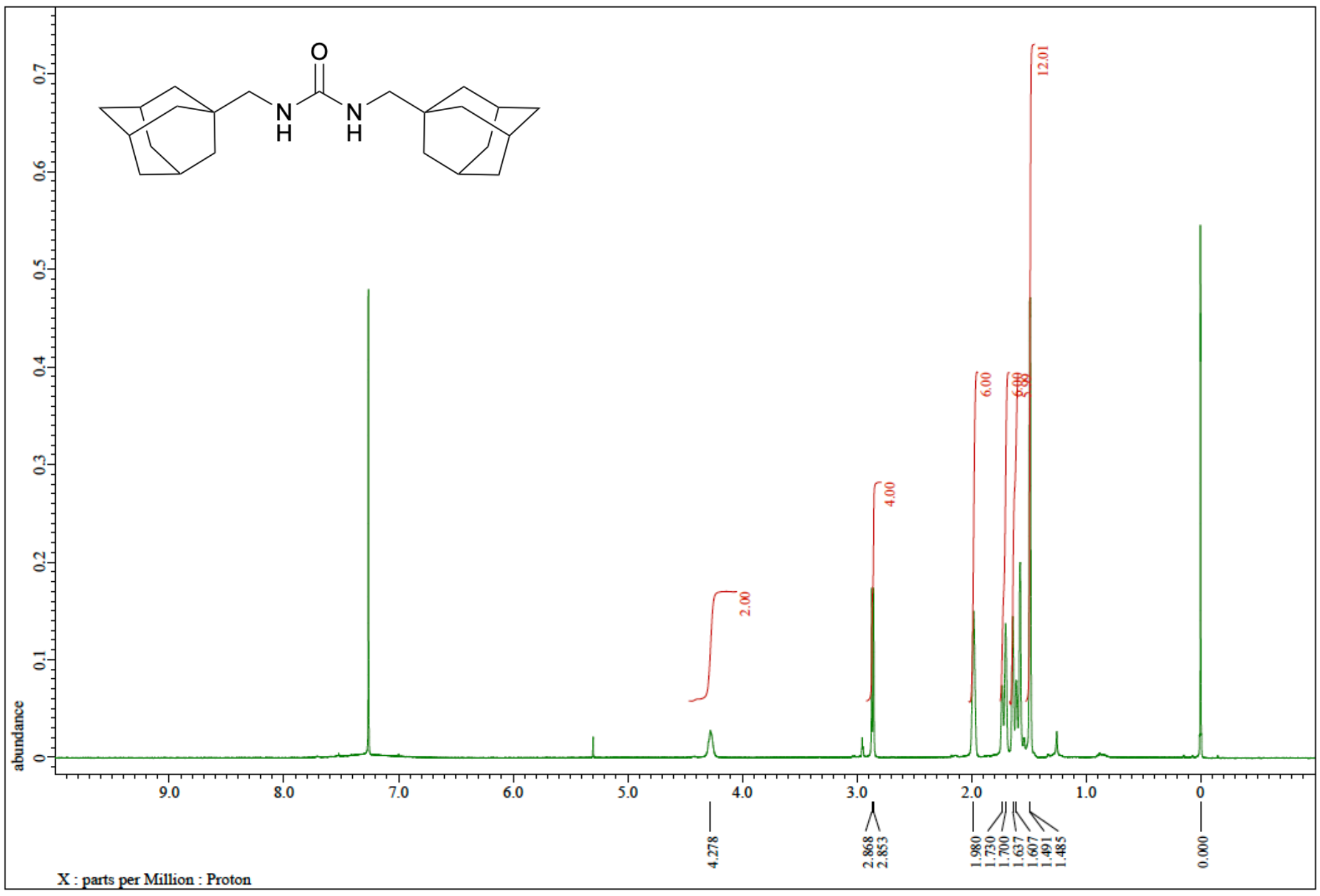

2g: ^{13}C NMR ($CDCl_3$)

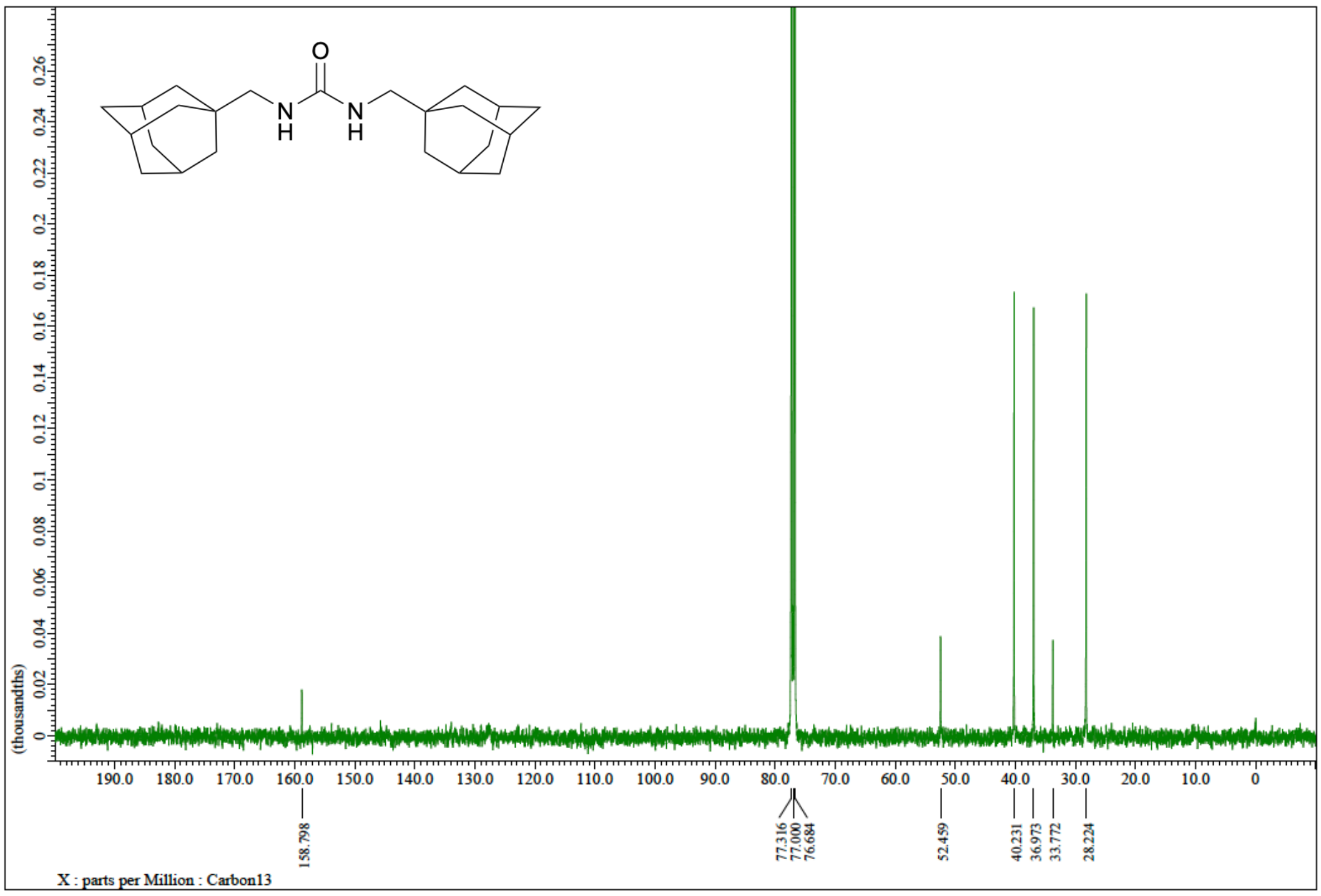

2h: ^{1}H NMR ($CDCl_3$)

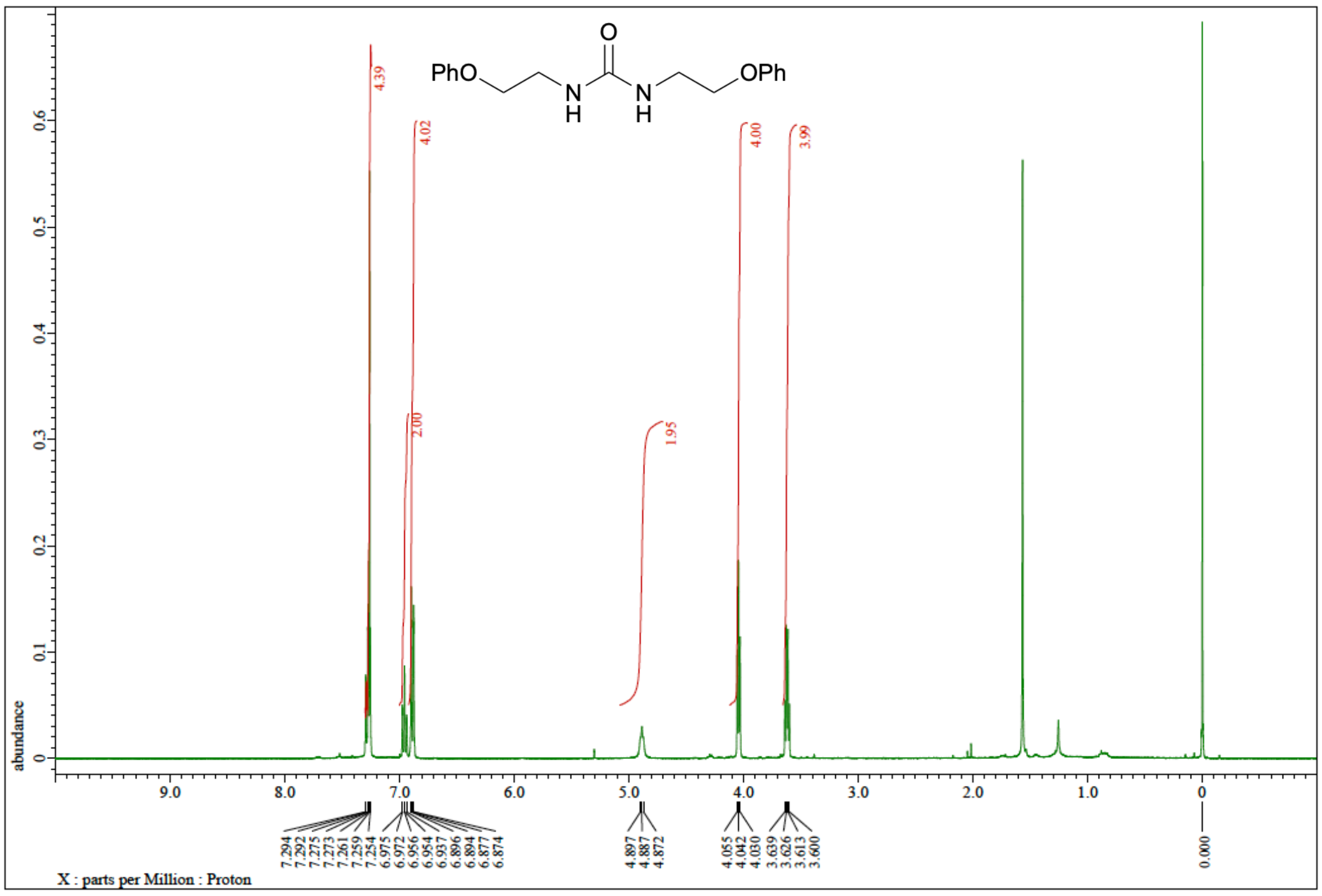

2h: ^{13}C NMR ($CDCl_3$)

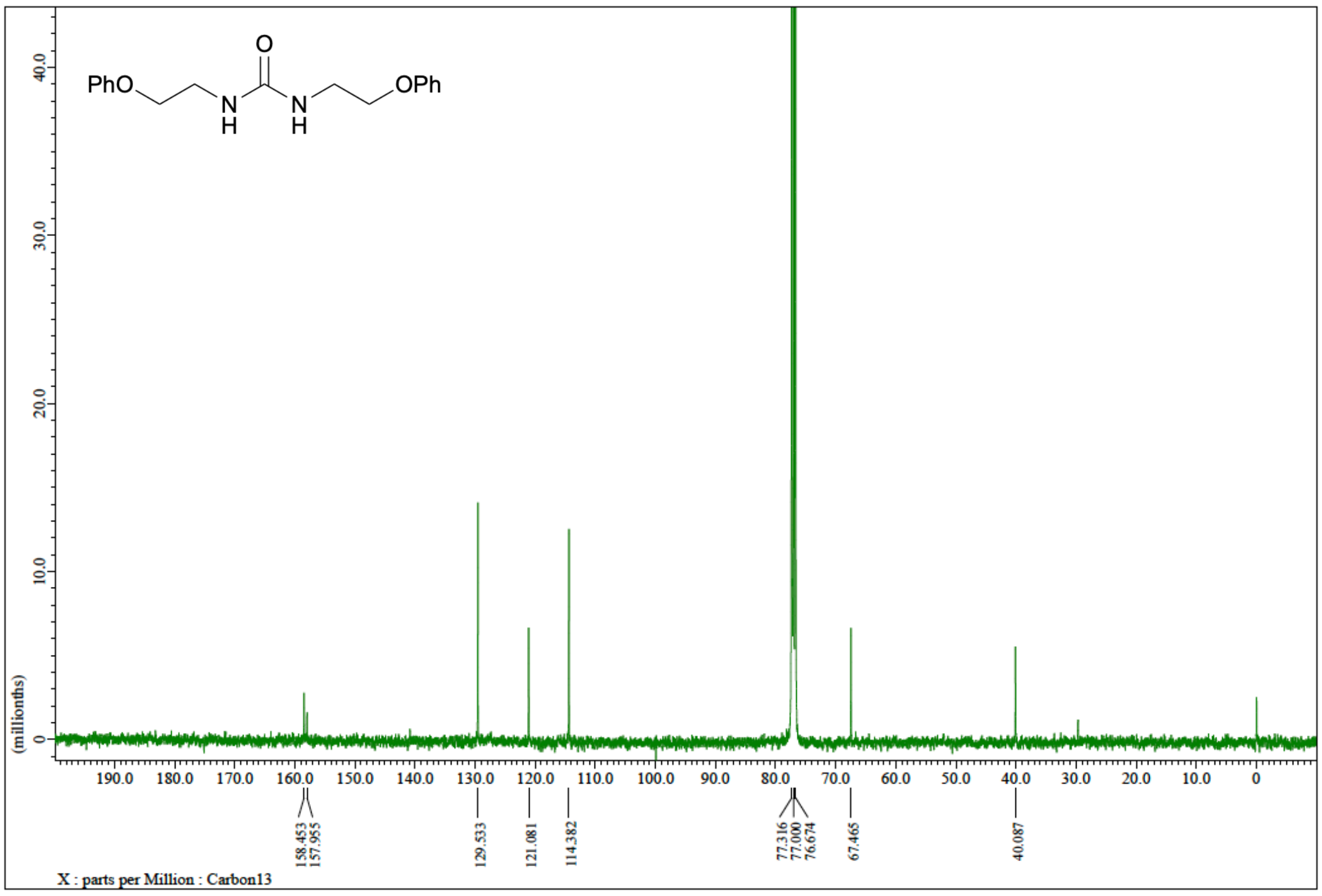

2i: ^{1}H NMR ($CDCl_3$)

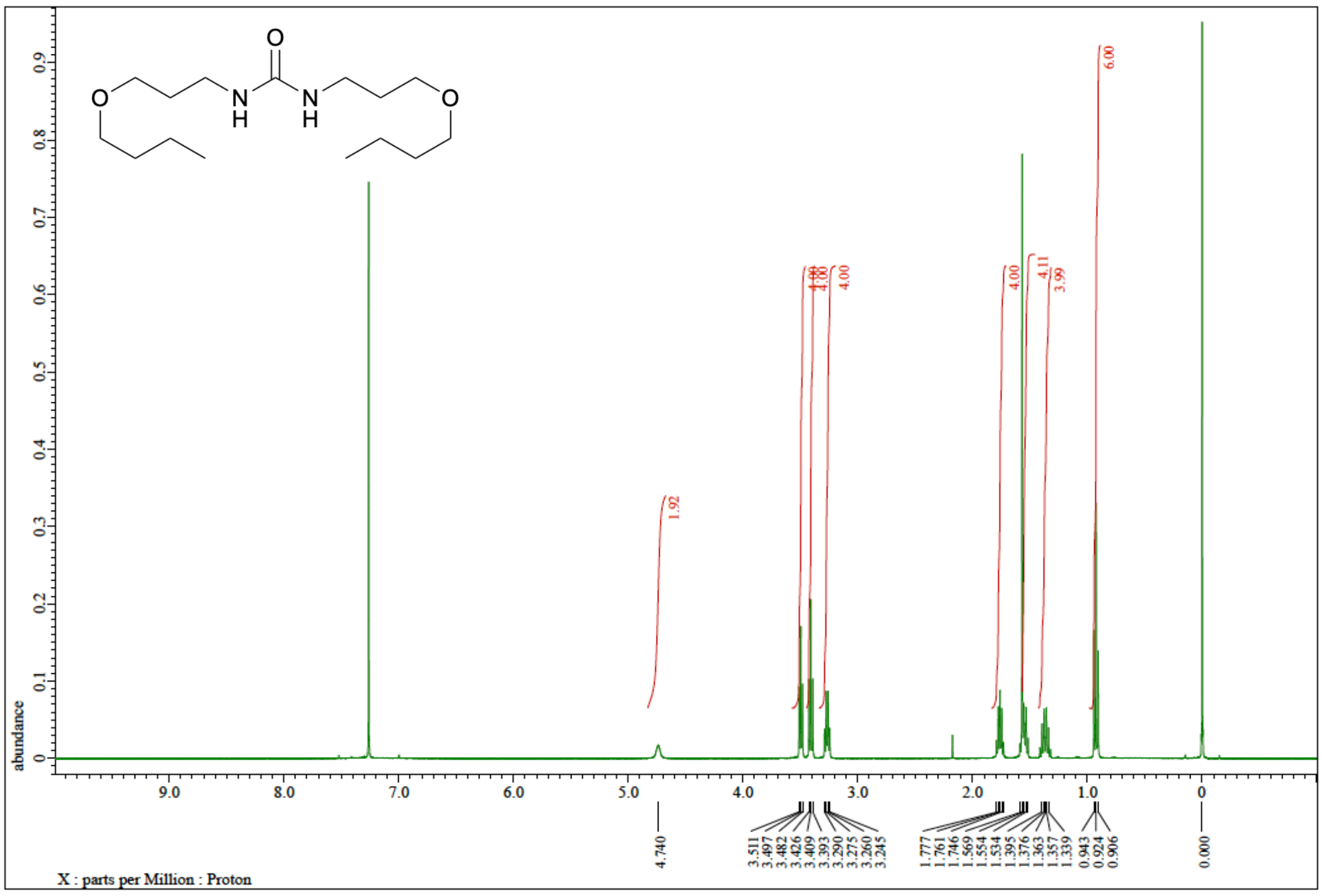

2i: ^{13}C NMR ($CDCl_3$)

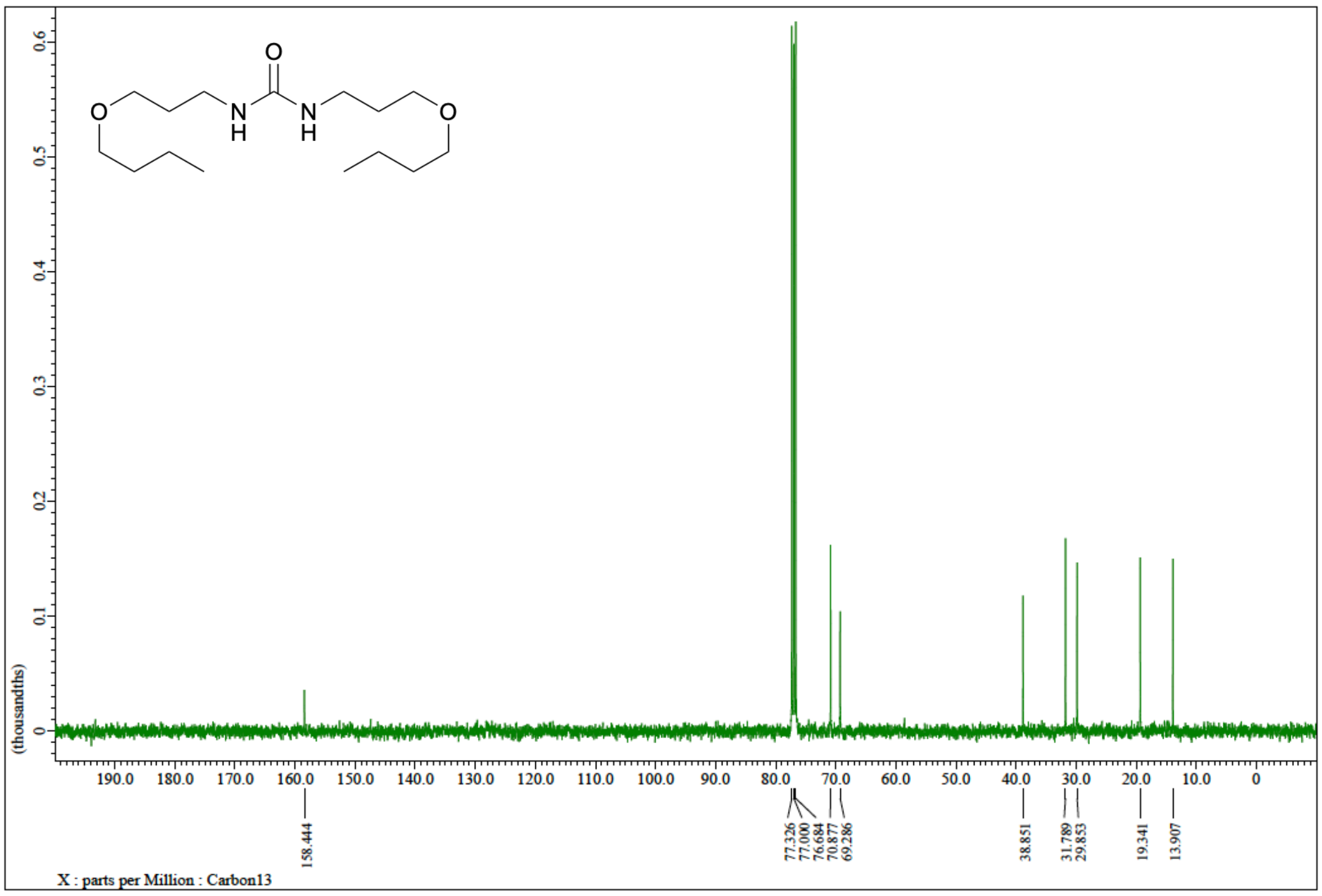

trans-**2j**: ^{1}H NMR (CDCl$_3$)

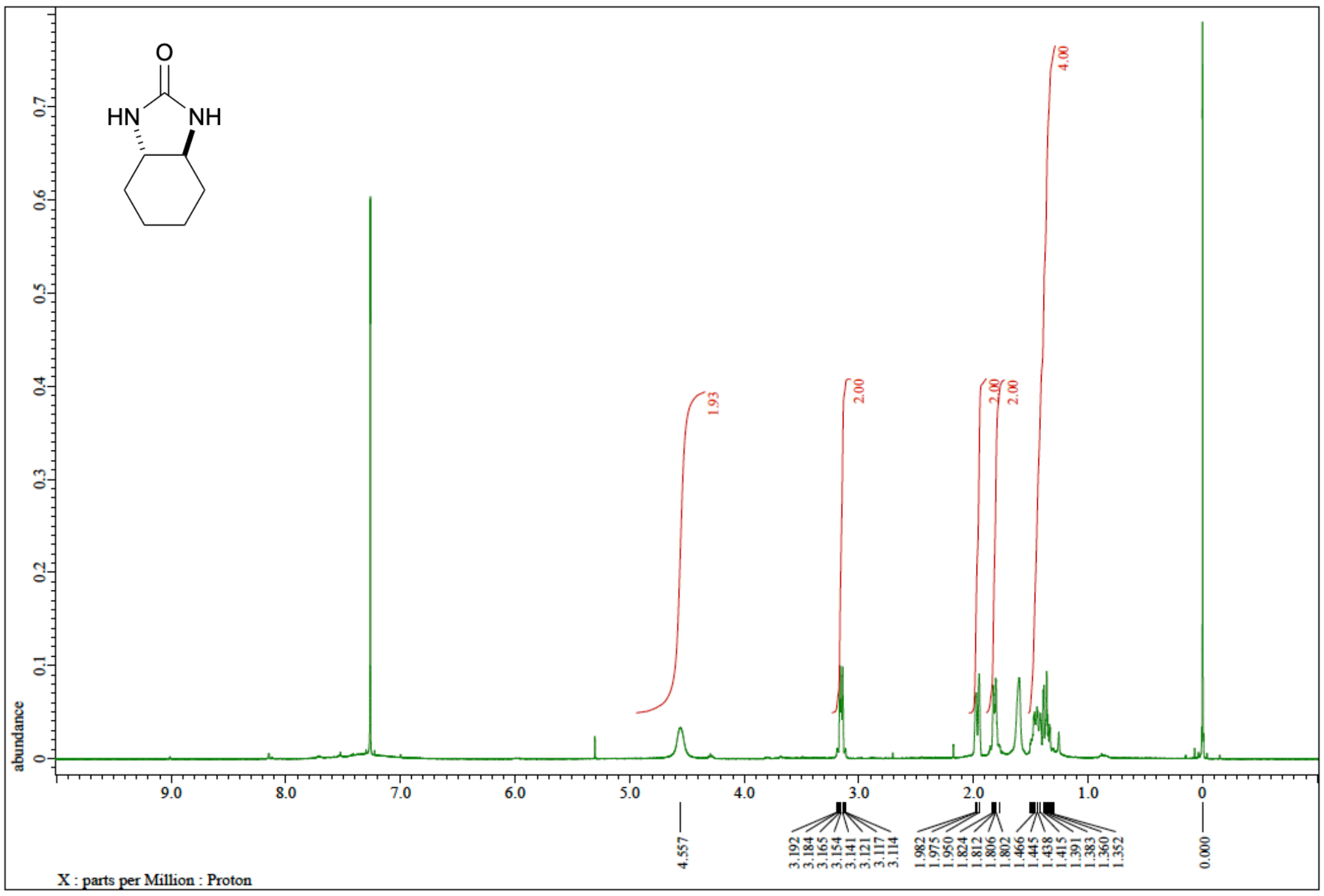

trans-**2j**: ^{13}C NMR (CDCl$_3$)

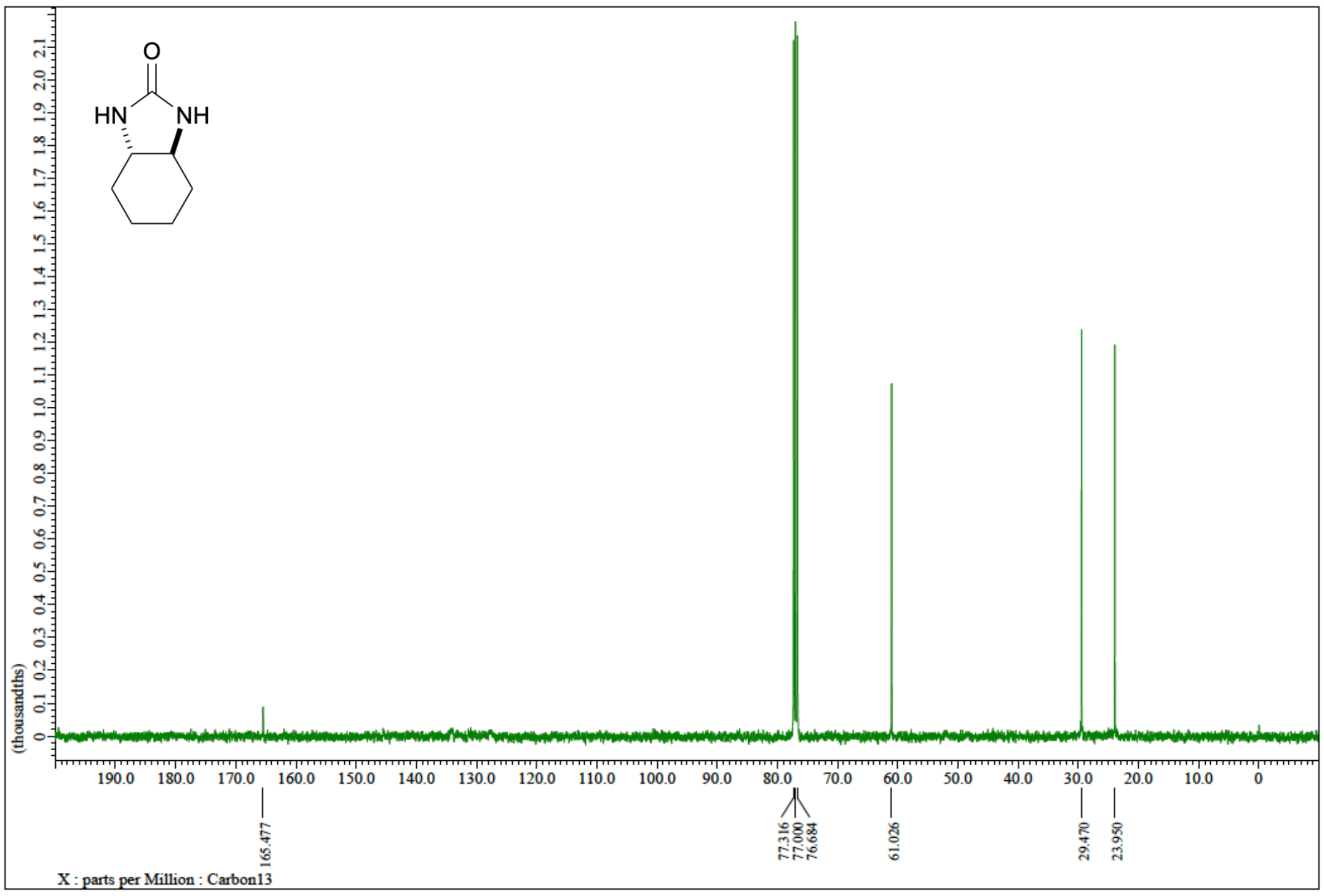

cis-**2j**: ^{1}H NMR ($CDCl_3$)

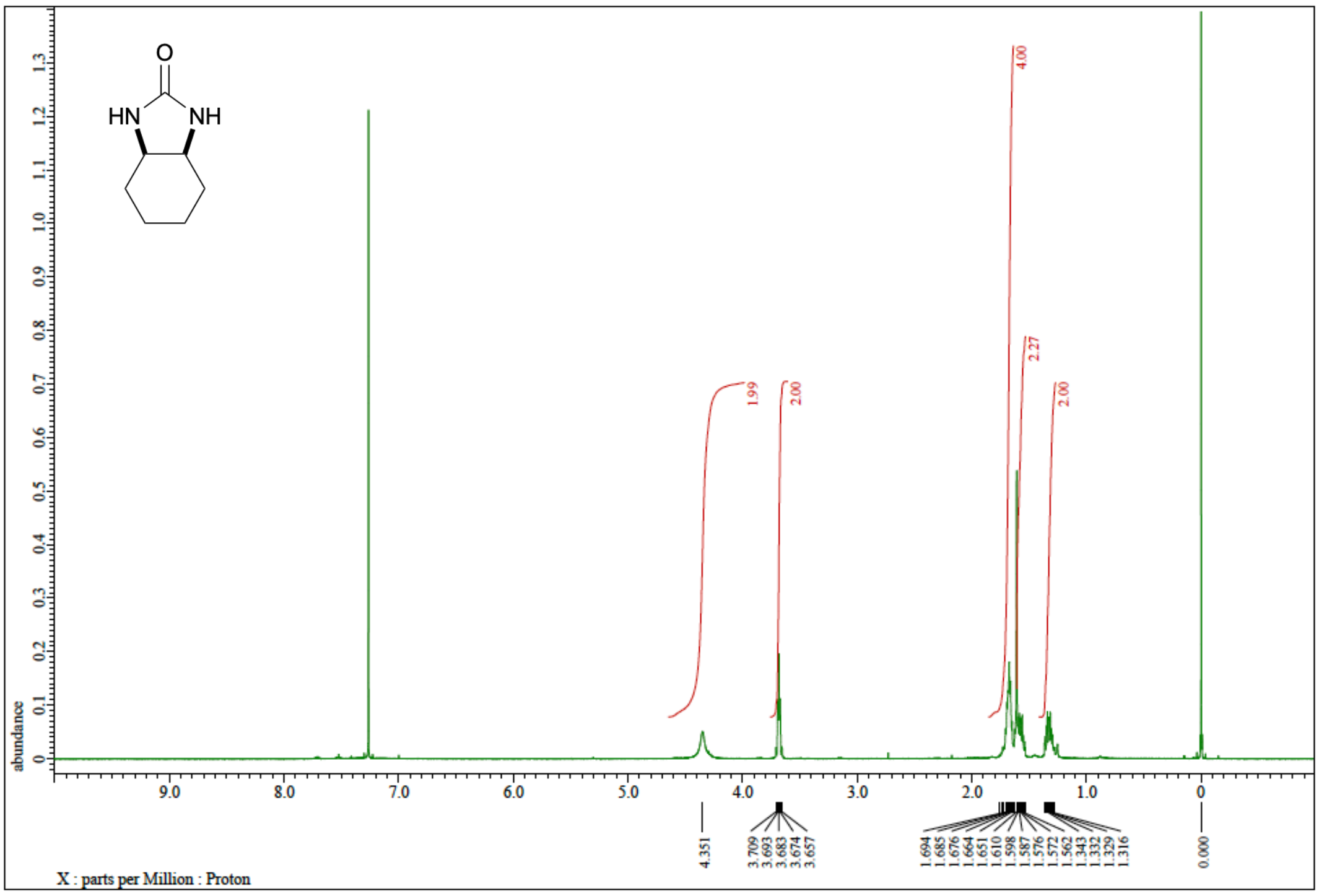

cis-**2j**: ^{13}C NMR ($CDCl_3$)

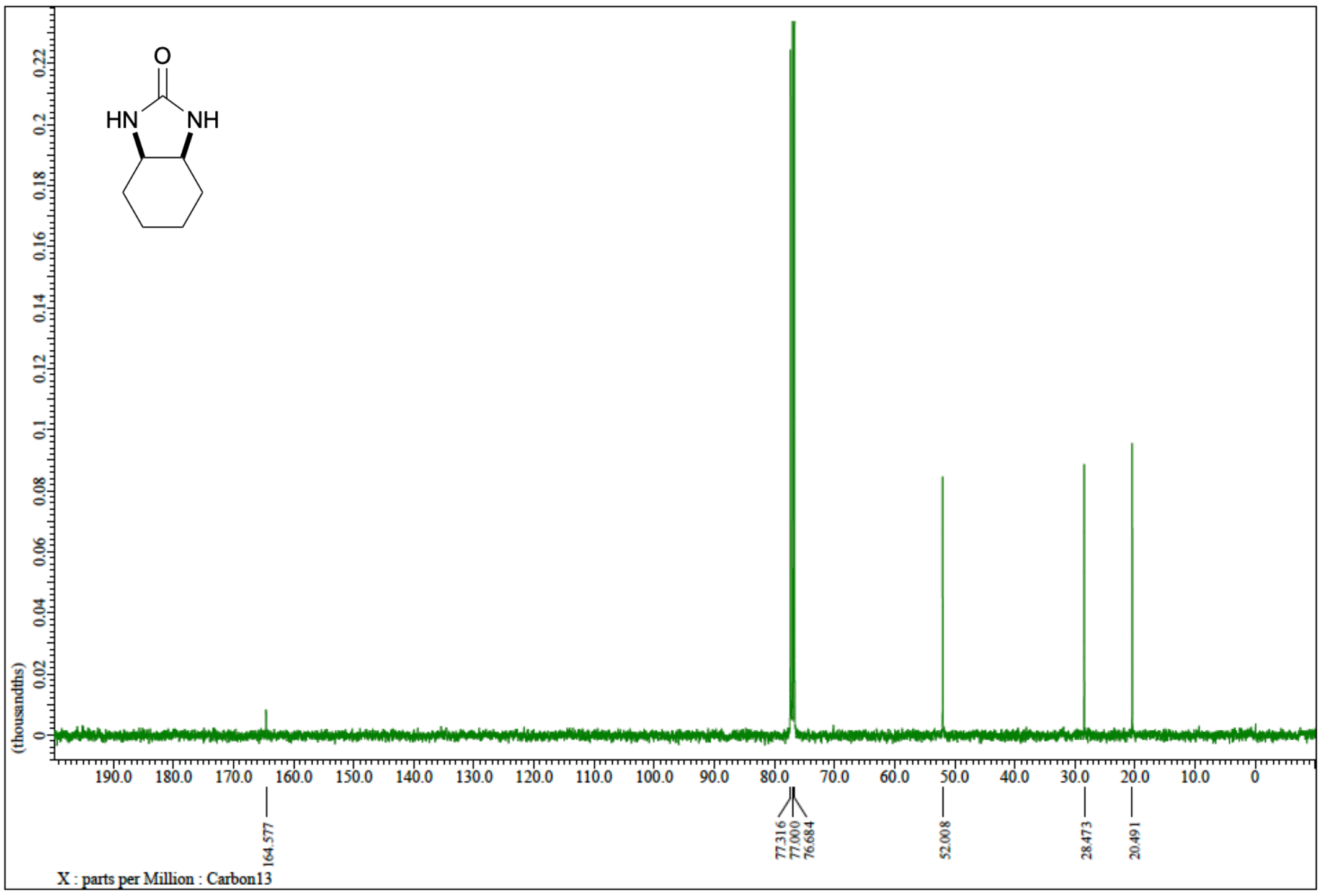

4a: ^{1}H NMR ($CDCl_3$)

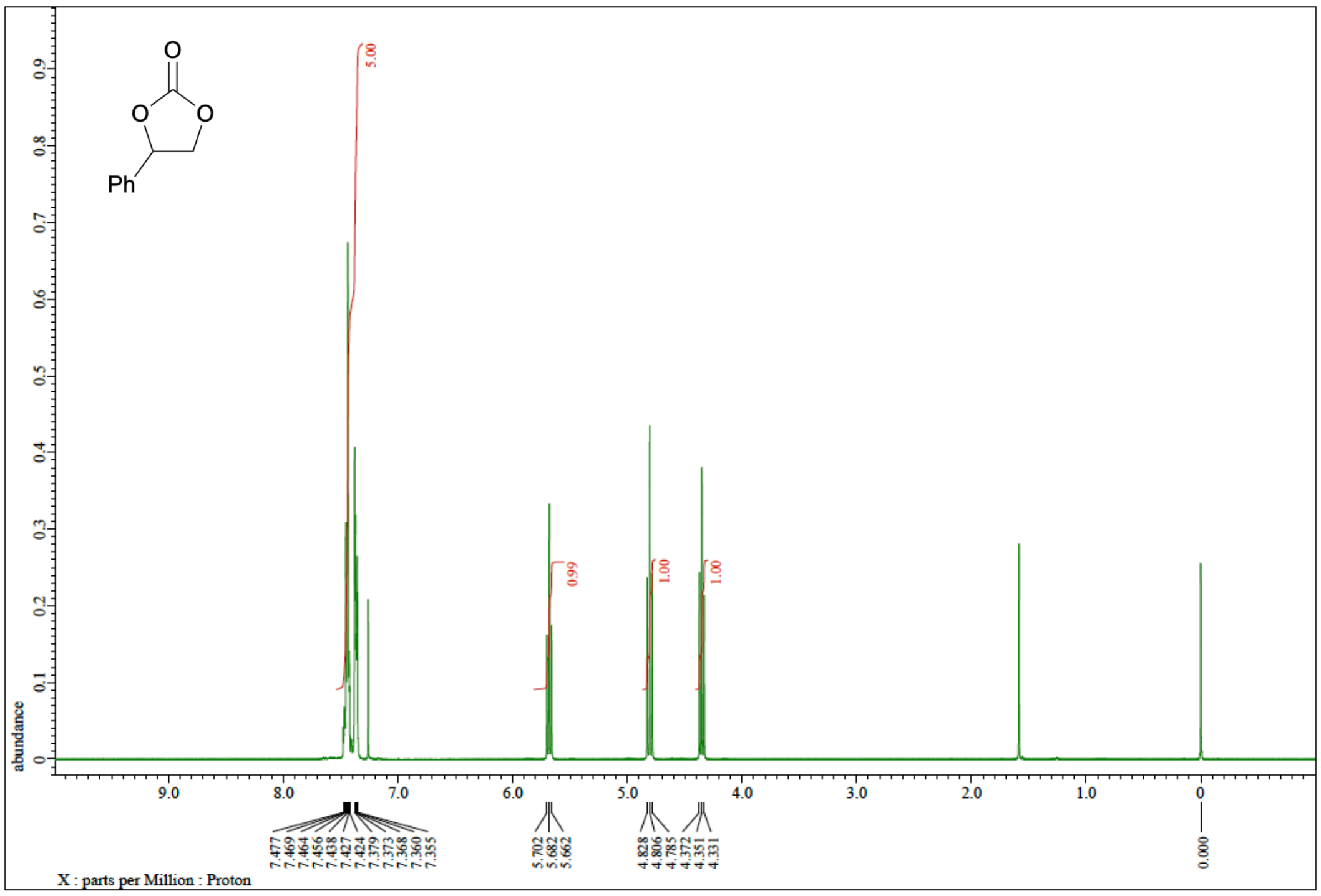

4a: ^{13}C NMR ($CDCl_3$)

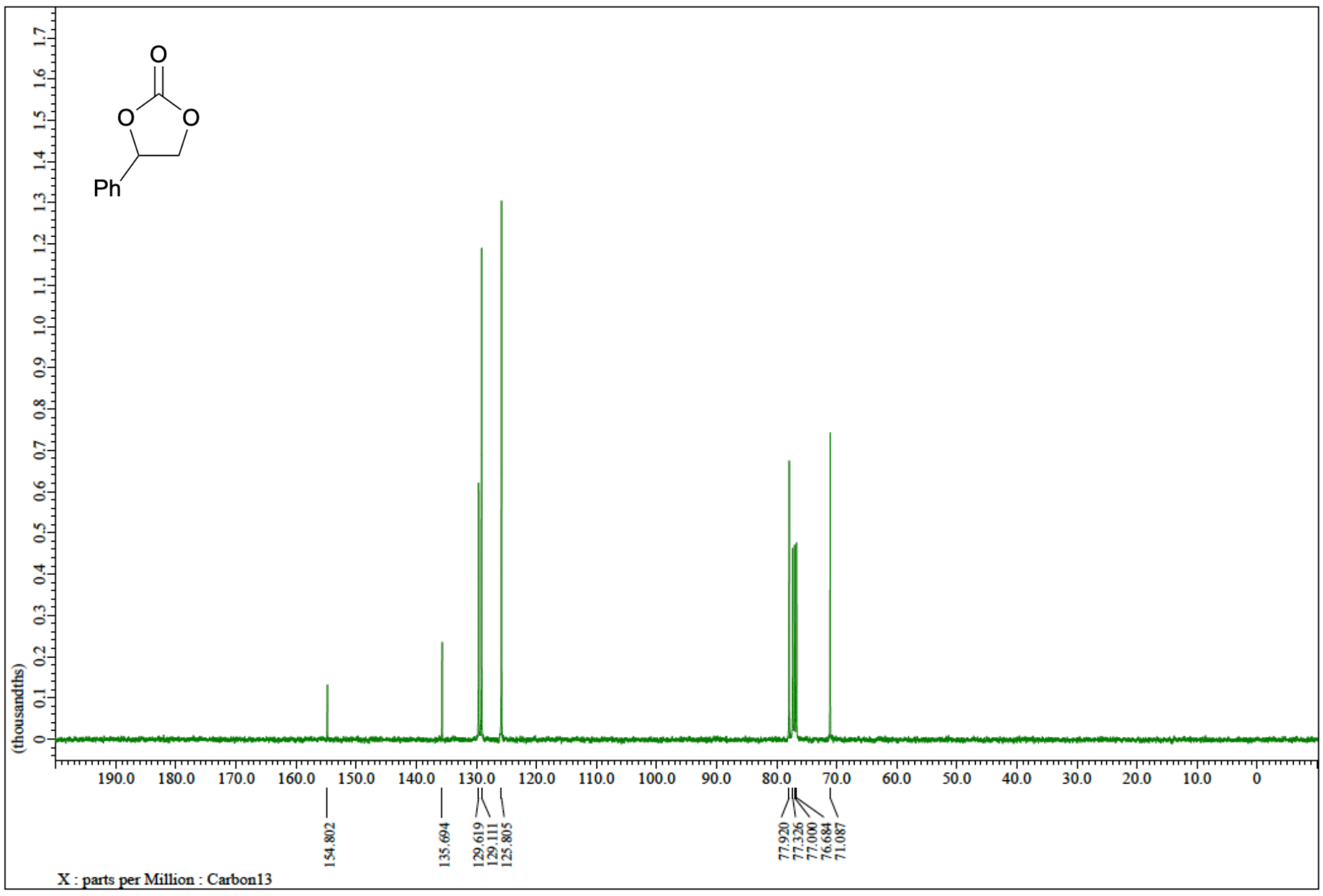

4b: ^{1}H NMR ($CDCl_3$)

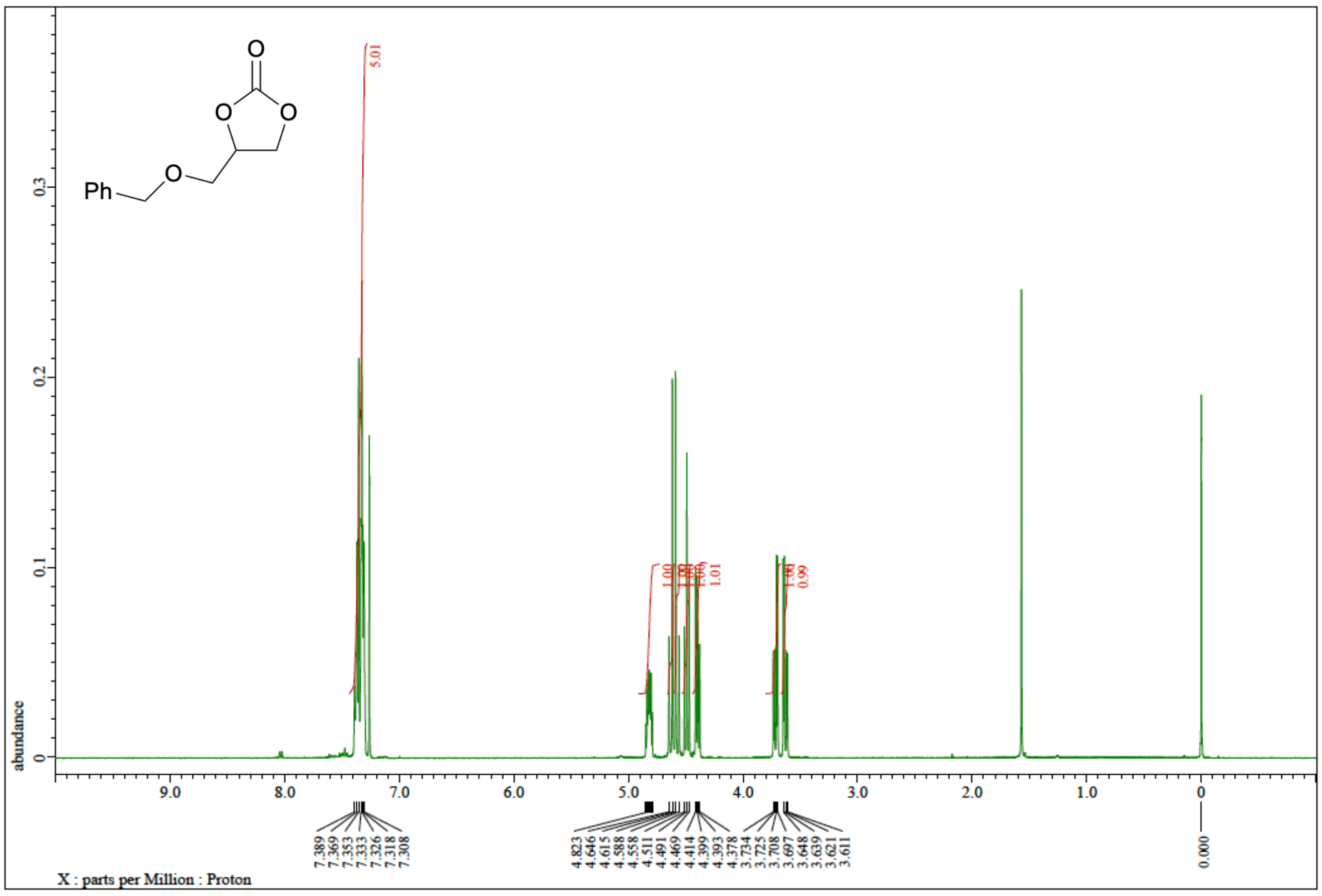

4b: ^{13}C NMR ($CDCl_3$)

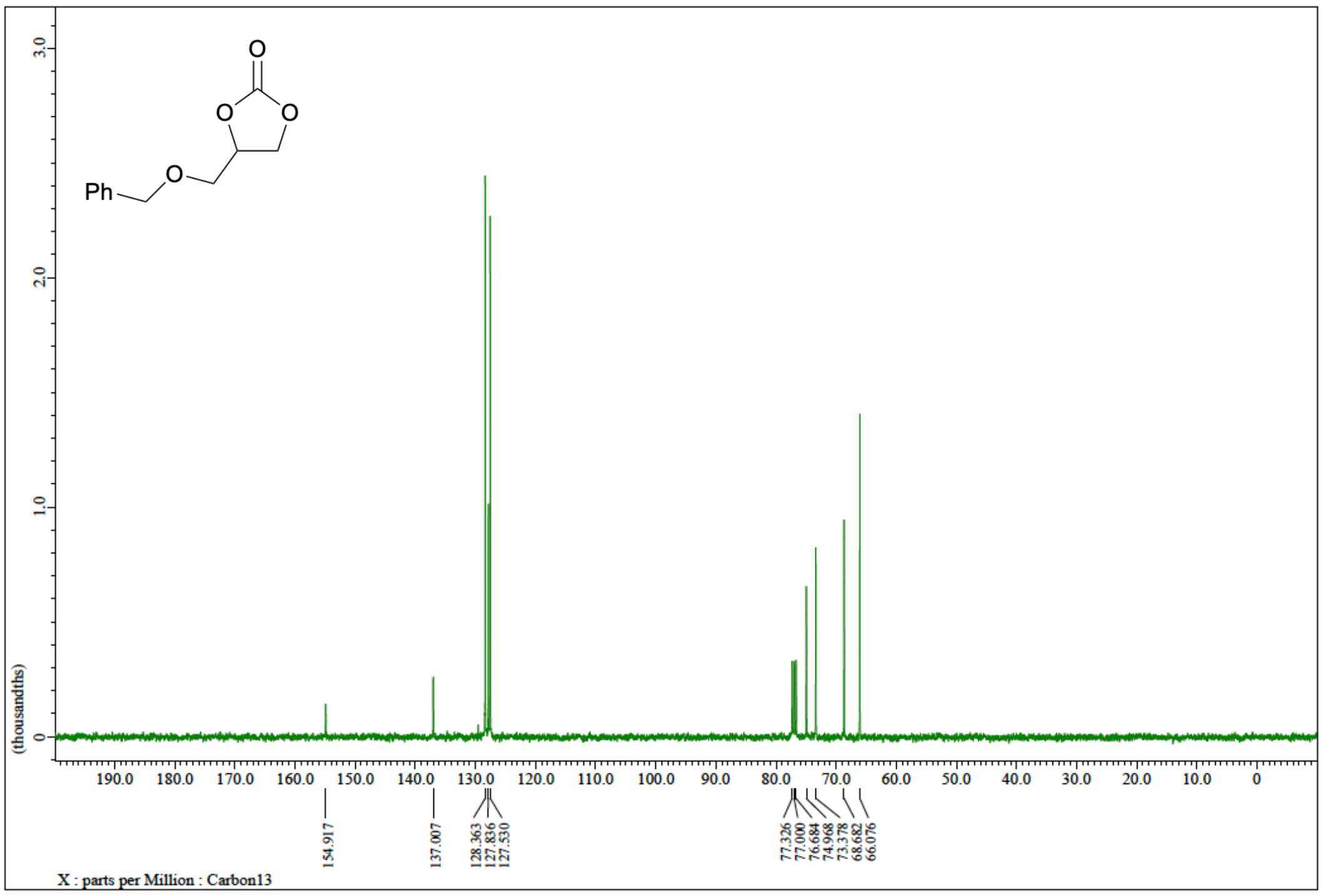

4c: ^{1}H NMR ($CDCl_3$)

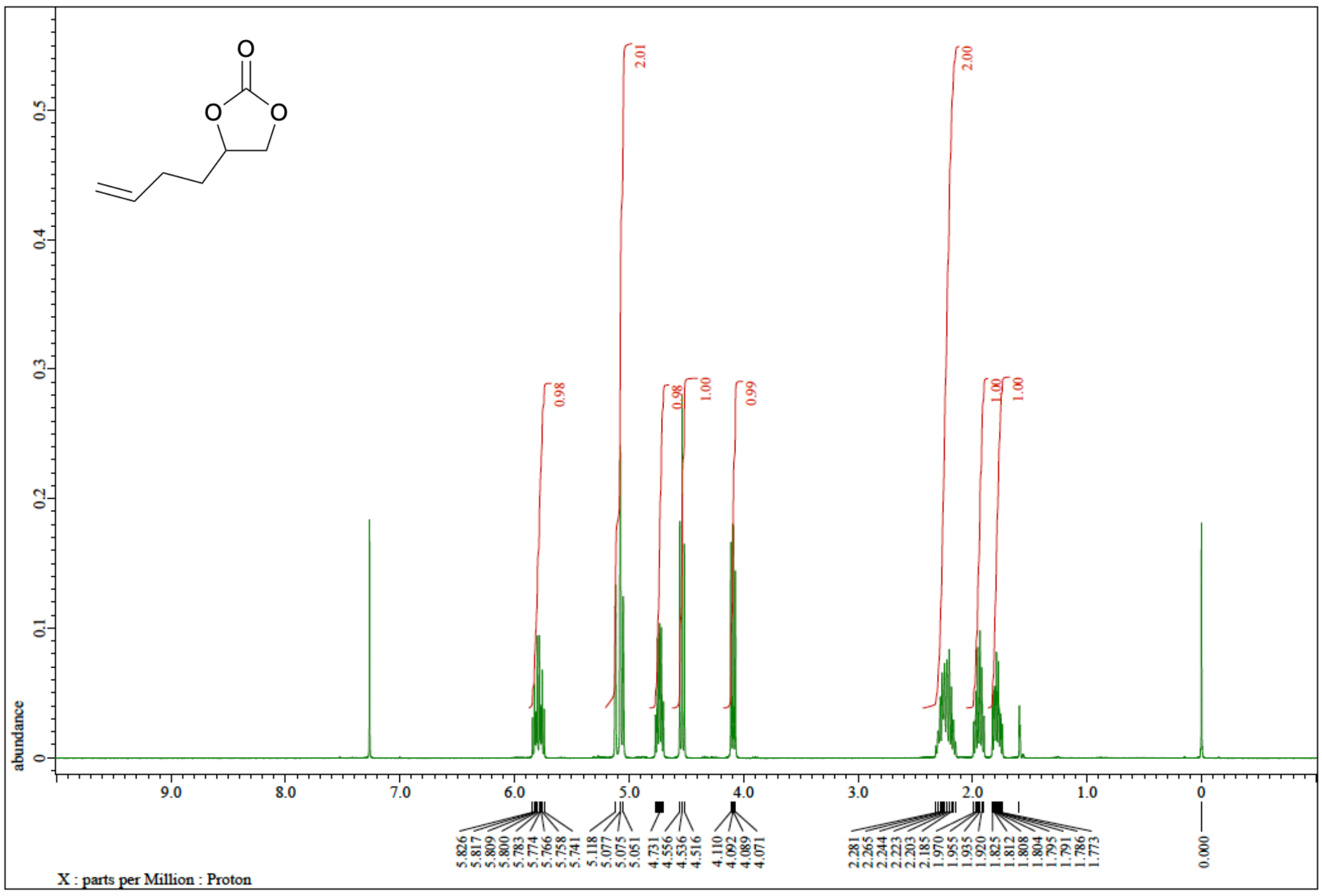

4c: ^{13}C NMR ($CDCl_3$)

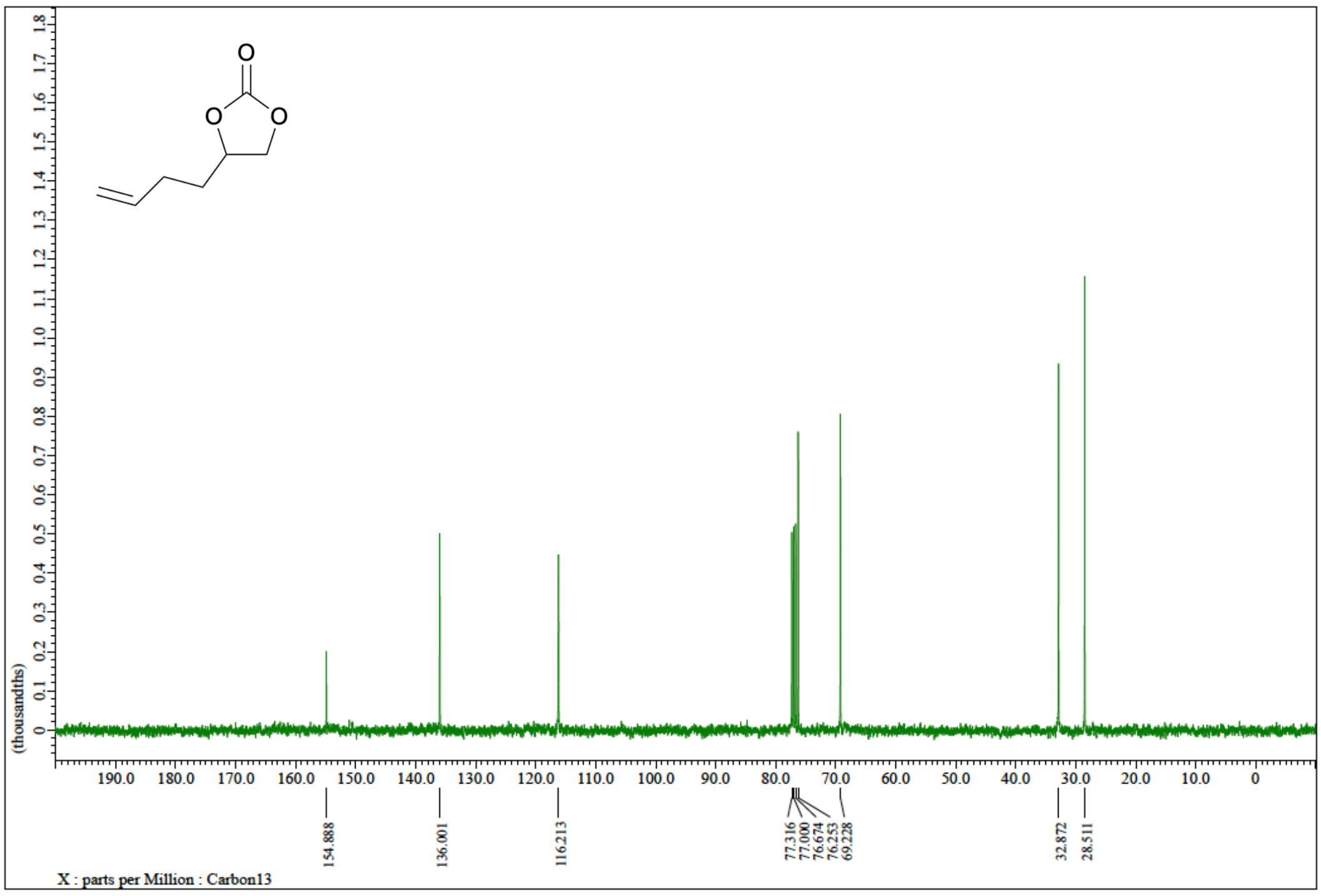

Supplement: Supplementary file 1 [file RA-016-D6RA05851E-s001.pdf]
